# Supplementary material for: Construction and validation of an RNA-binding protein-associated prognostic model for colorectal cancer
Source: PeerJ. 2021 Apr 5;9:e11219. doi: 10.7717/peerj.11219 (PMC8029696; doi:10.7717/peerj.11219)
Supplement: Supplemental Information 1 [file peerj-09-11219-s001.docx]

**Table S1.** Characteristics of TCGA colorectal cancer cohort and GEO dataset.

|  |  | **TCGA** | **GEO** |
| --- | --- | --- | --- |
| **Age(years)** |  | 66.20±12.85 | 65.55±12.69 |
| **Gender** | Female | 281 | 301 |
|  | Male | 315 | 419 |
| **Stage** | Stage Ⅰ | 107 | 31 |
|  | Stage Ⅱ | 223 | 311 |
|  | Stage Ⅲ | 177 | 309 |
|  | Stage Ⅳ | 89 | 69 |
| **T (Tumor)** | T1 | 18 | 11 |
|  | T2 | 105 | 48 |
|  | T3 | 407 | 535 |
|  | T4 | 66 | 126 |
| **N (Lymph Node)** | N0 | 354 | 356 |
|  | N1 | 150 | 261 |
|  | N2 | 116 | 97 |
|  | N3 | 0 | 6 |
| **M (Metastasis)** | M0 | 455 | 650 |
|  | M1 | 87 | 70 |
|  | Mx | 54 | 68 |
