# Supplementary material for: Construction and validation of an RNA-binding protein-associated prognostic model for colorectal cancer
Source: PeerJ. 2021 Apr 5;9:e11219. doi: 10.7717/peerj.11219 (PMC8029696; doi:10.7717/peerj.11219)

	pvalue	Hazard ratio
ZC3H12C	0.020	0.807(0.674−0.967)
PNLDC1	0.031	1.227(1.019−1.476)
LUZP4	<0.001	1.531(1.246−1.882)
RRS1	0.011	1.484(1.095−2.011)
HEXIM1	0.038	1.371(1.018−1.847)
SRRM3	0.037	1.659(1.031−2.671)
PPARGC1A	0.046	0.809(0.657−0.996)
PPARGC1B	0.006	0.594(0.410−0.862)
RBPMS2	0.040	1.310(1.013−1.695)
BRCA1	0.028	0.653(0.447−0.955)
CELF4	<0.001	1.676(1.265−2.220)
AEN	0.023	1.351(1.043−1.752)
LRRFIP2	0.043	0.707(0.506−0.989)
EIF4E3	0.033	0.675(0.471−0.969)
NOVA1	0.002	1.327(1.108−1.589)

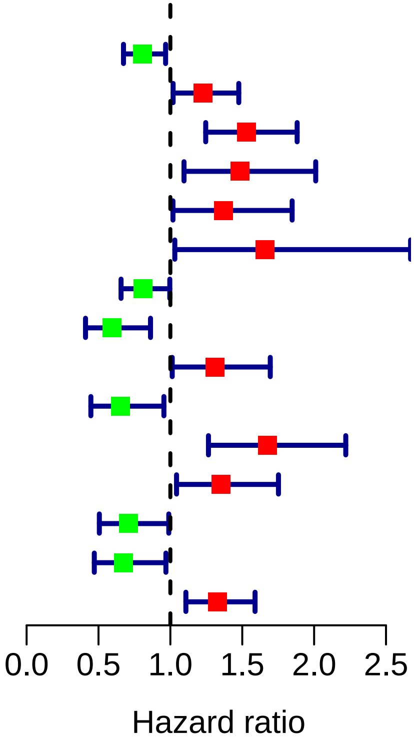

Supplement: Supplemental Information 6 [file peerj-09-11219-s006.zip › raw data/21.uniCox/forest.pdf]
