# Supplementary figures and images for: Construction and validation of an RNA-binding protein-associated prognostic model for colorectal cancer
Source: PeerJ. 2021 Apr 5;9:e11219. doi: 10.7717/peerj.11219 (PMC8029696; doi:10.7717/peerj.11219)

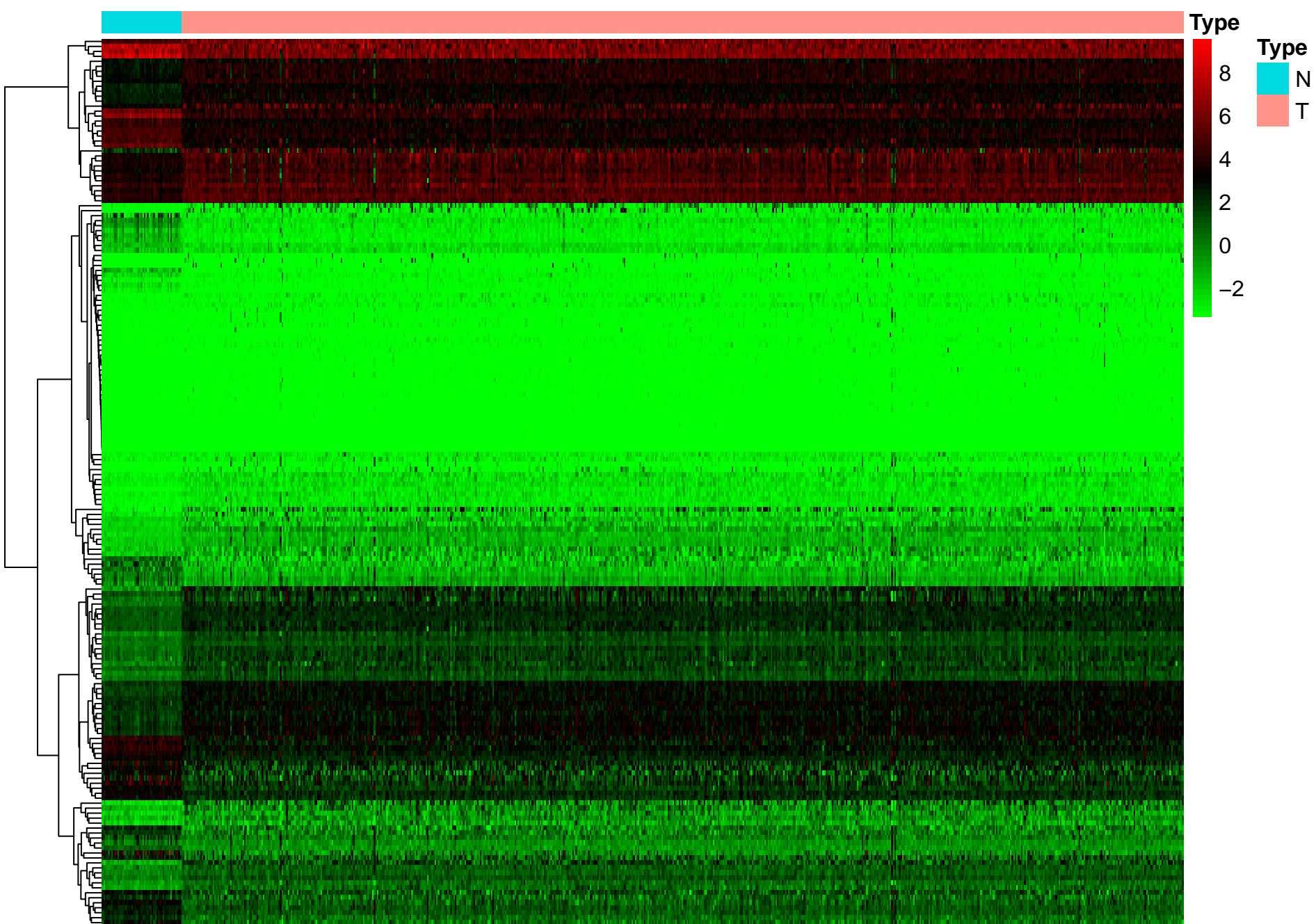

Supplement: Supplemental Information 6 [file peerj-09-11219-s006.zip › raw data/10.diff/heatmap.pdf]

# Volcano

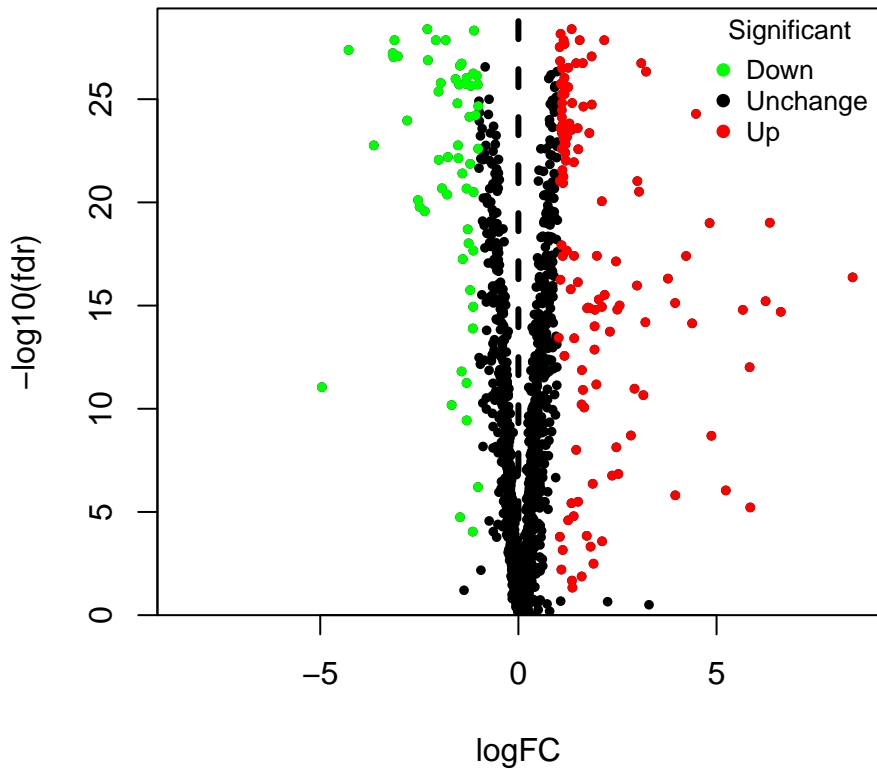

Supplement: Supplemental Information 6 [file peerj-09-11219-s006.zip › raw data/10.diff/vol.pdf]

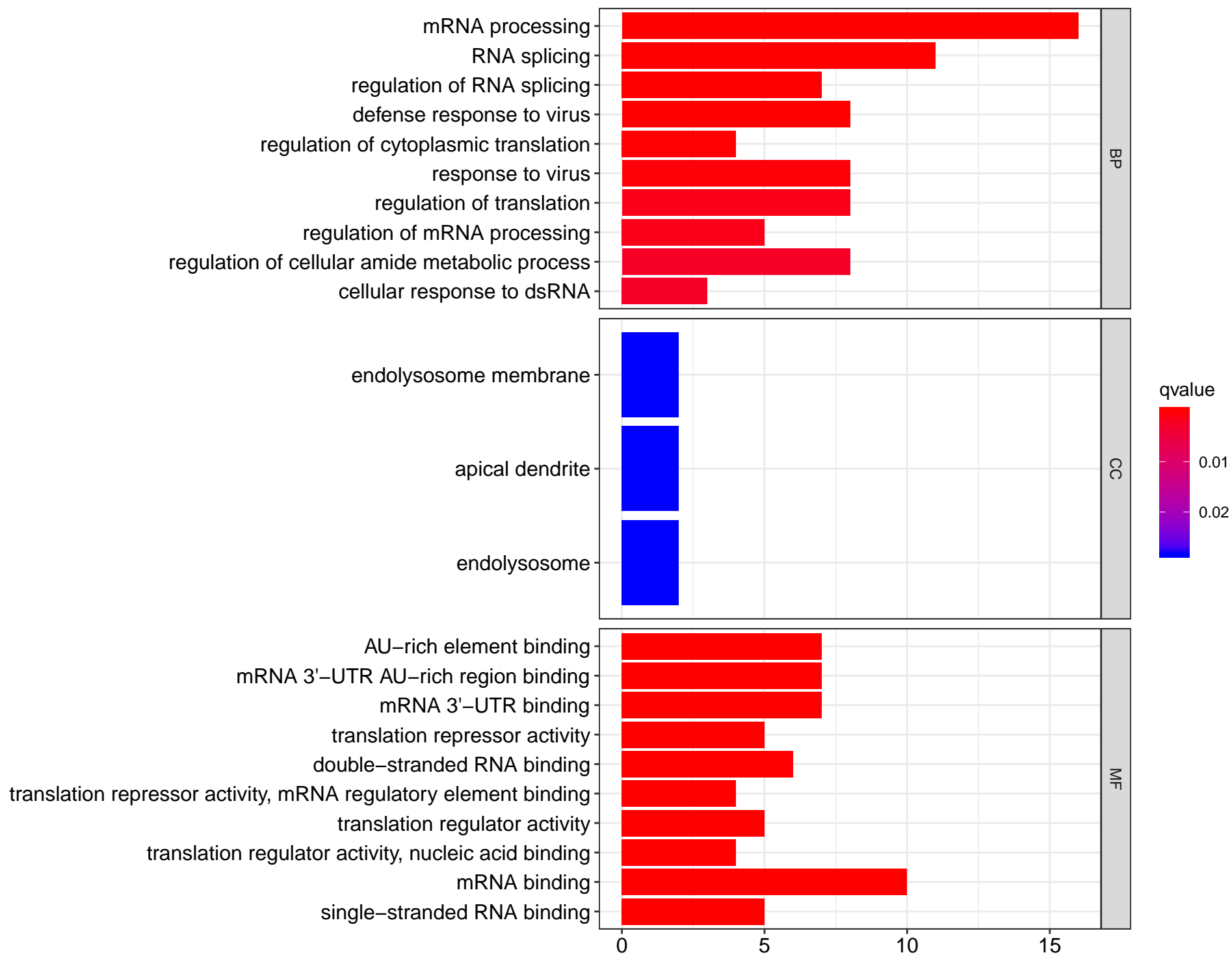

Supplement: Supplemental Information 6 [file peerj-09-11219-s006.zip › raw data/11.GO/down.barplot.pdf]

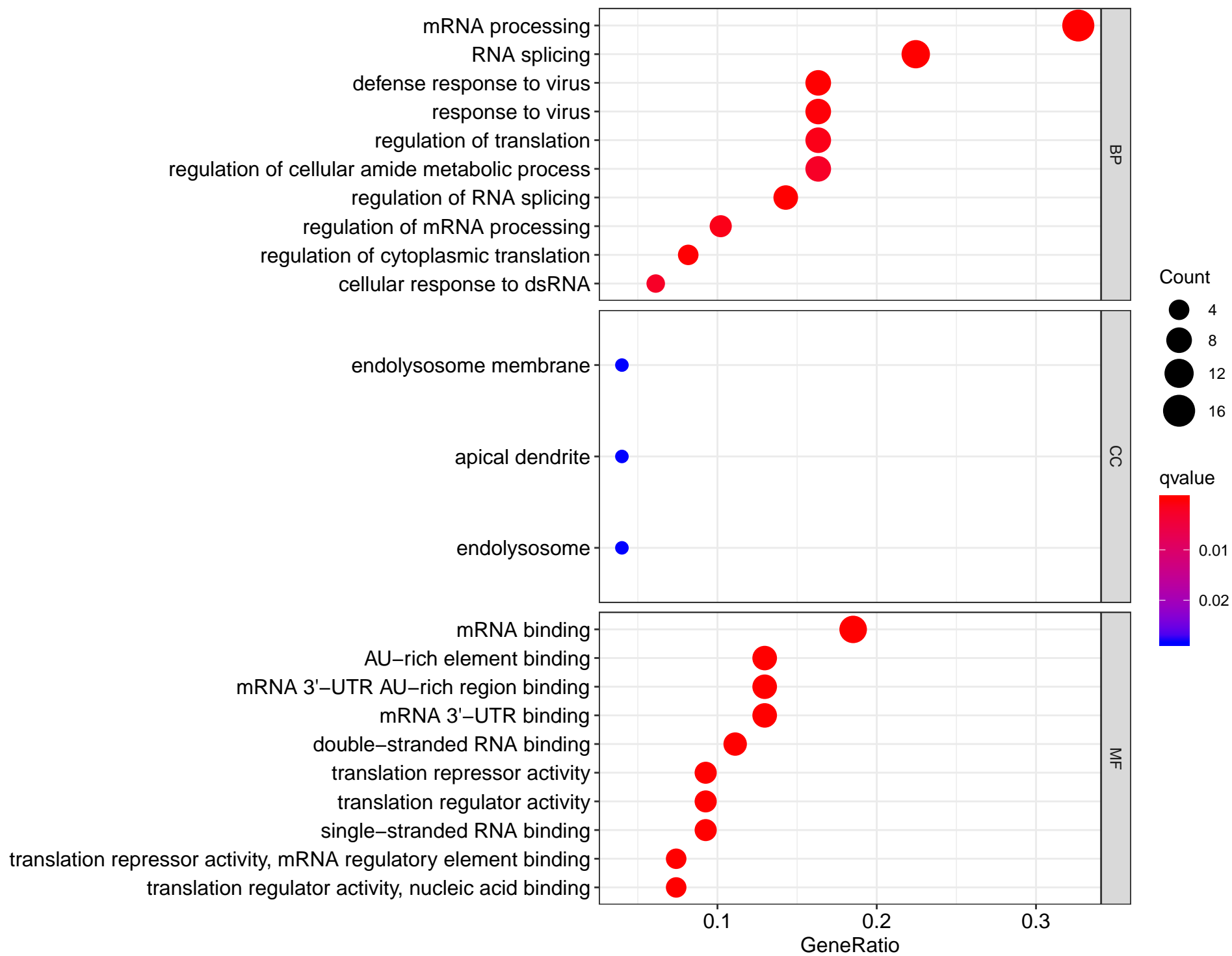

Supplement: Supplemental Information 6 [file peerj-09-11219-s006.zip › raw data/11.GO/down.bubble.pdf]

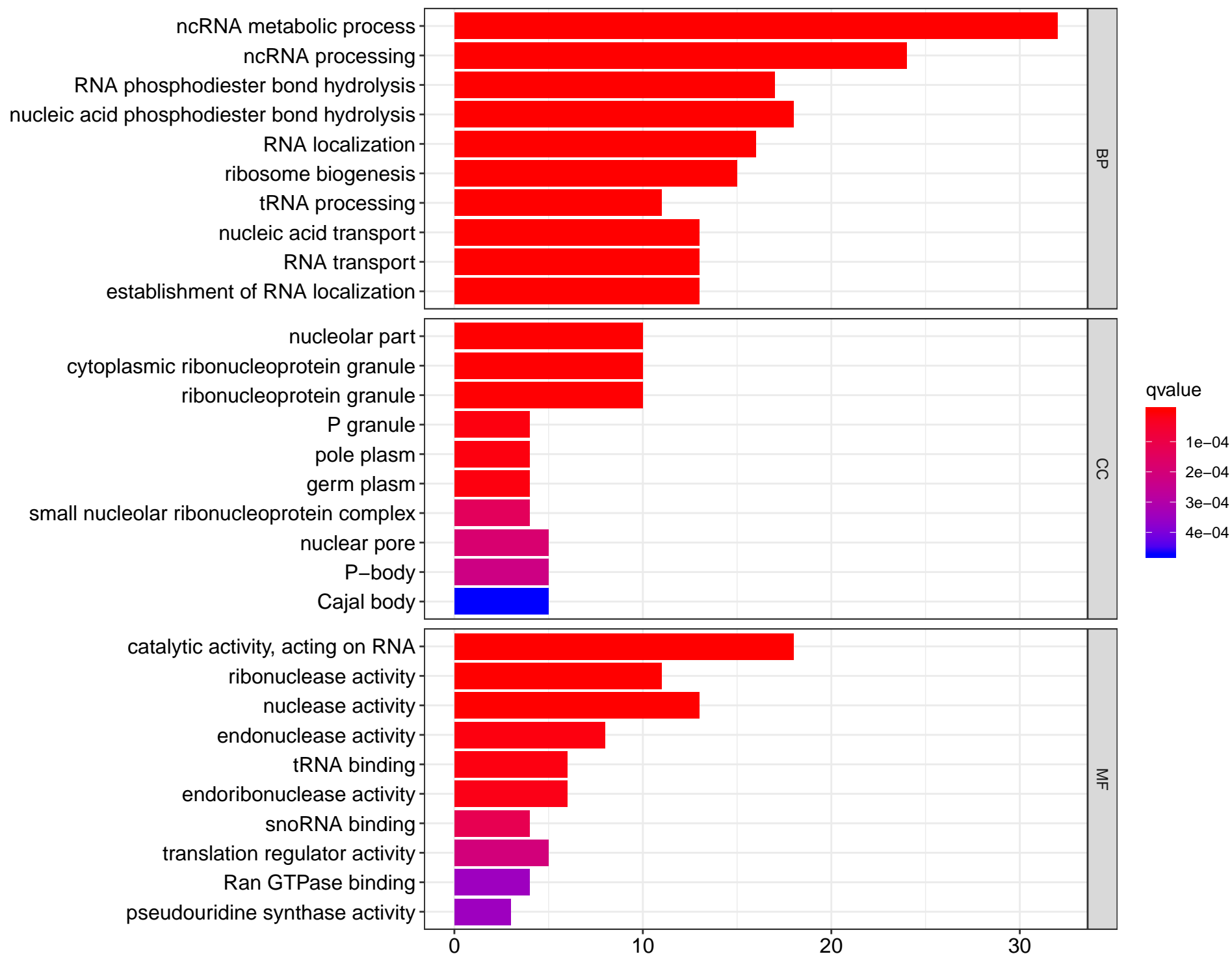

Supplement: Supplemental Information 6 [file peerj-09-11219-s006.zip › raw data/11.GO/up.barplot.pdf]

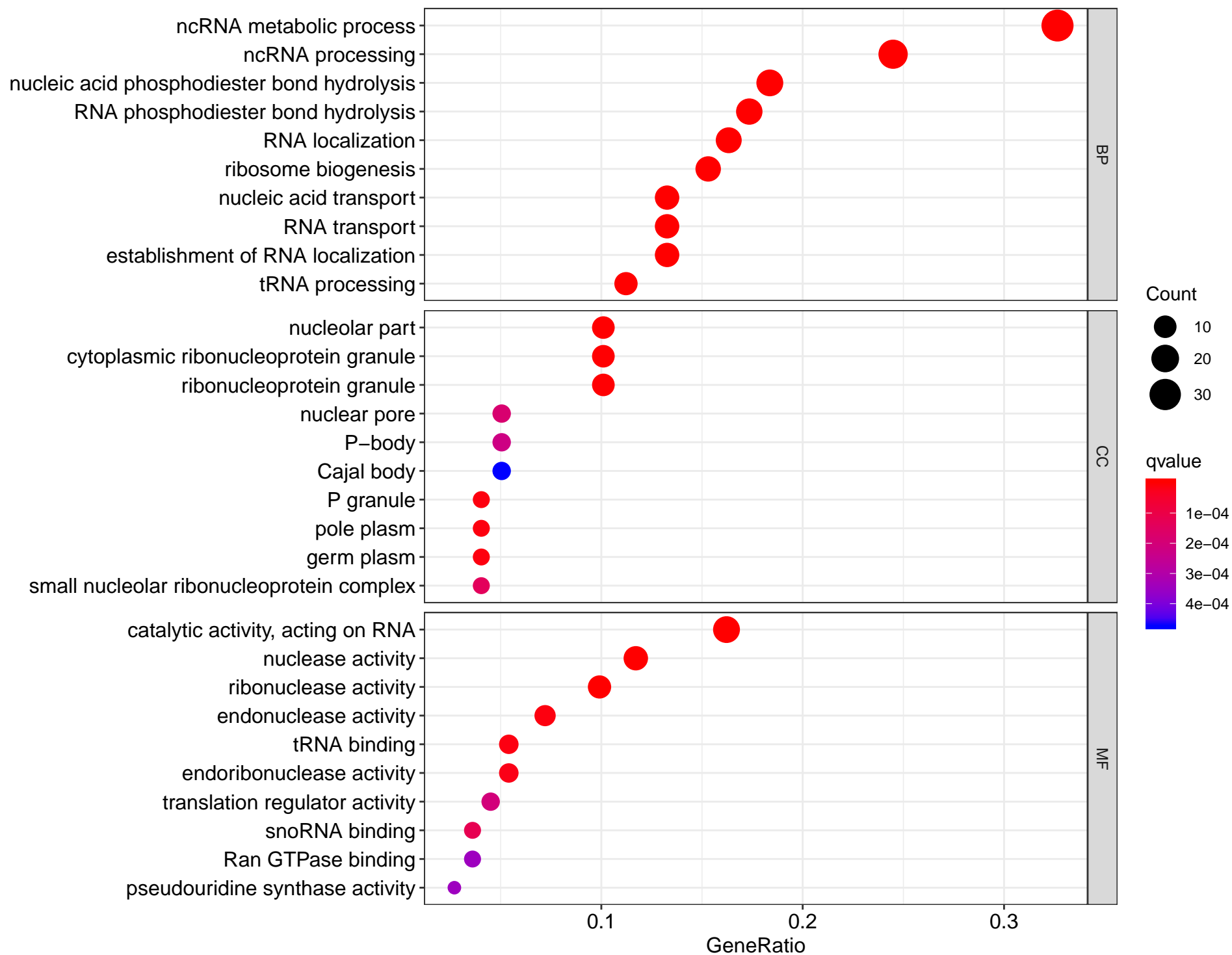

Supplement: Supplemental Information 6 [file peerj-09-11219-s006.zip › raw data/11.GO/up.bubble.pdf]

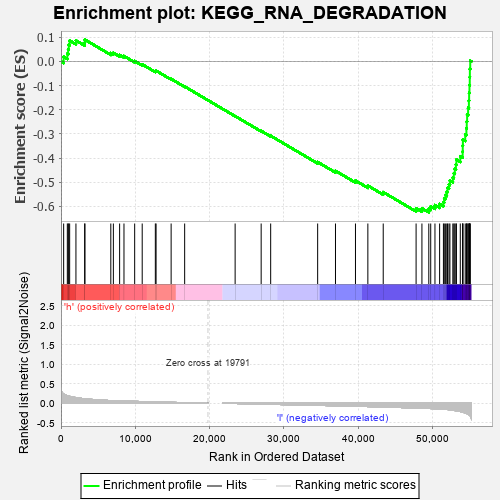

Supplement: Supplemental Information 6 [file peerj-09-11219-s006.zip › raw data/12.KEGG/25.GSEA/TOP5-up and down/1down.png]

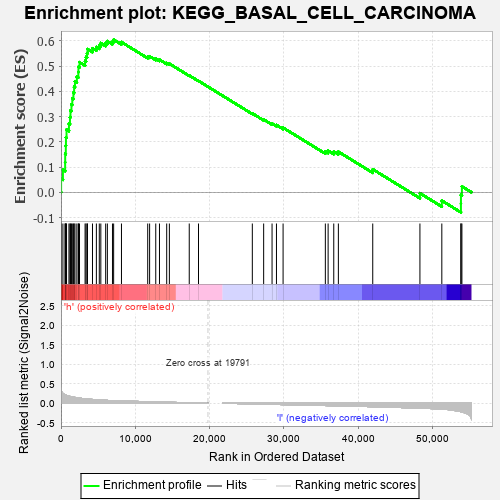

Supplement: Supplemental Information 6 [file peerj-09-11219-s006.zip › raw data/12.KEGG/25.GSEA/TOP5-up and down/1up.png]

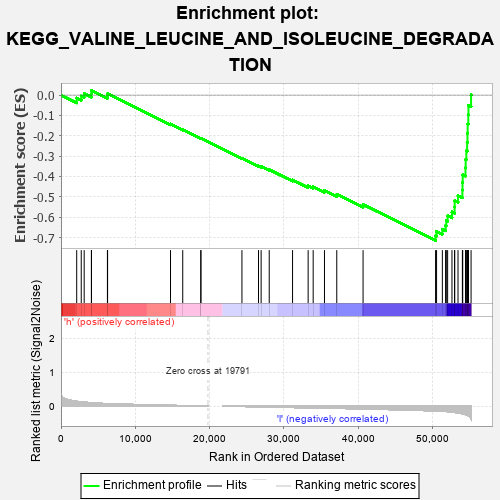

Supplement: Supplemental Information 6 [file peerj-09-11219-s006.zip › raw data/12.KEGG/25.GSEA/TOP5-up and down/2down.png]

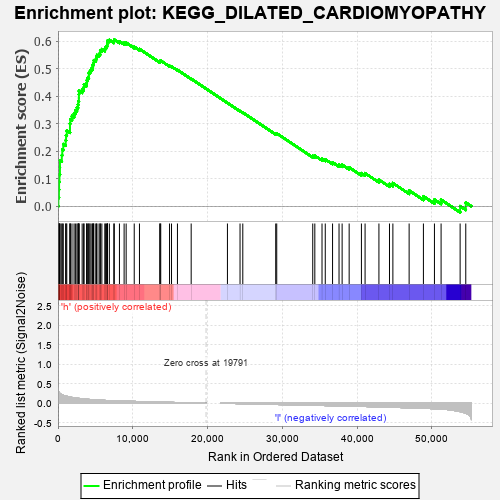

Supplement: Supplemental Information 6 [file peerj-09-11219-s006.zip › raw data/12.KEGG/25.GSEA/TOP5-up and down/2up.png]

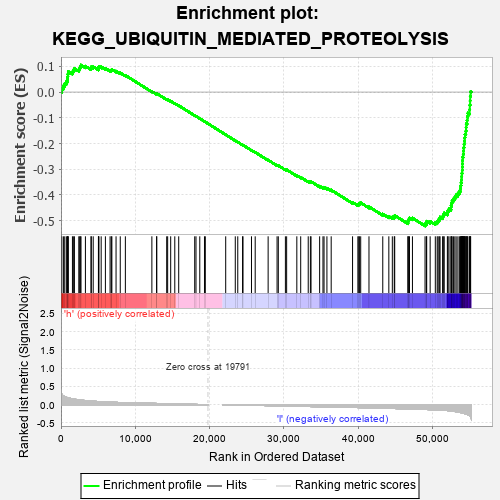

Supplement: Supplemental Information 6 [file peerj-09-11219-s006.zip › raw data/12.KEGG/25.GSEA/TOP5-up and down/3down.png]

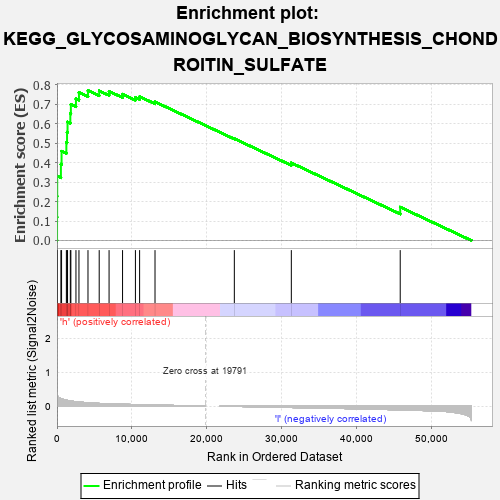

Supplement: Supplemental Information 6 [file peerj-09-11219-s006.zip › raw data/12.KEGG/25.GSEA/TOP5-up and down/3up.png]

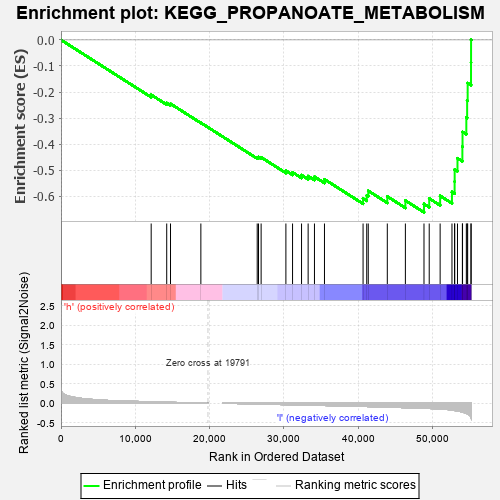

Supplement: Supplemental Information 6 [file peerj-09-11219-s006.zip › raw data/12.KEGG/25.GSEA/TOP5-up and down/4down.png]

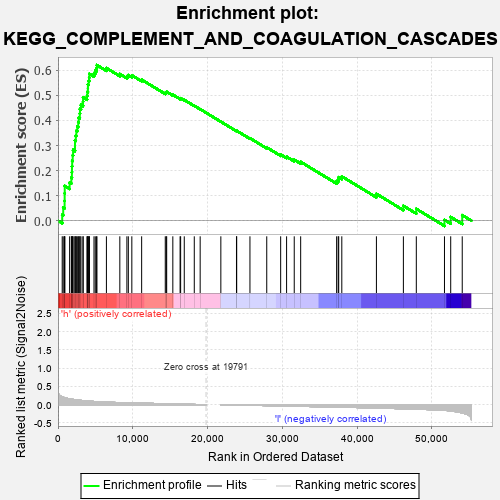

Supplement: Supplemental Information 6 [file peerj-09-11219-s006.zip › raw data/12.KEGG/25.GSEA/TOP5-up and down/4up.png]

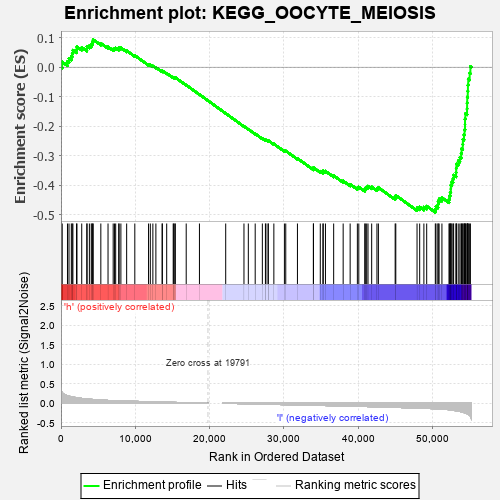

Supplement: Supplemental Information 6 [file peerj-09-11219-s006.zip › raw data/12.KEGG/25.GSEA/TOP5-up and down/5down.png]

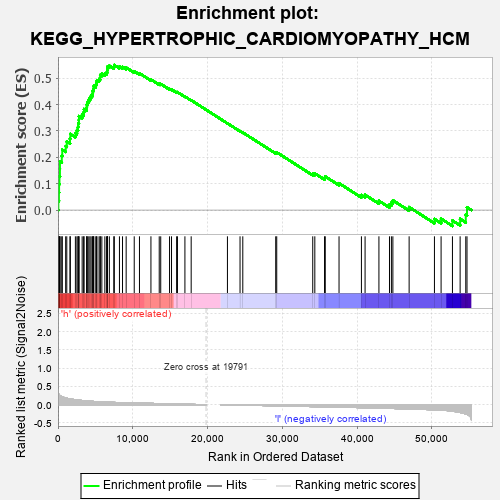

Supplement: Supplemental Information 6 [file peerj-09-11219-s006.zip › raw data/12.KEGG/25.GSEA/TOP5-up and down/5up.png]

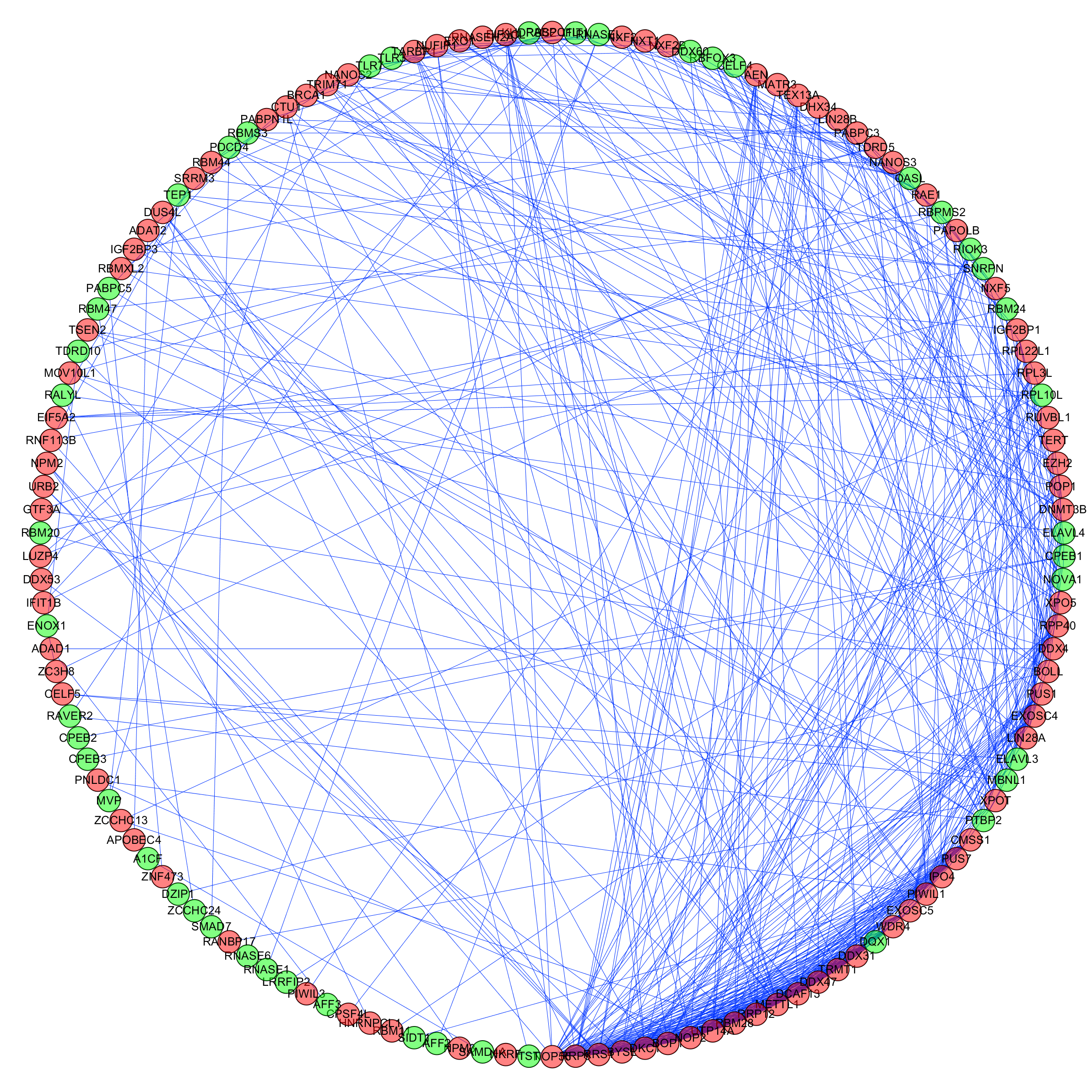

Supplement: Supplemental Information 6 [file peerj-09-11219-s006.zip › raw data/15.cytoscape/ppi.png]

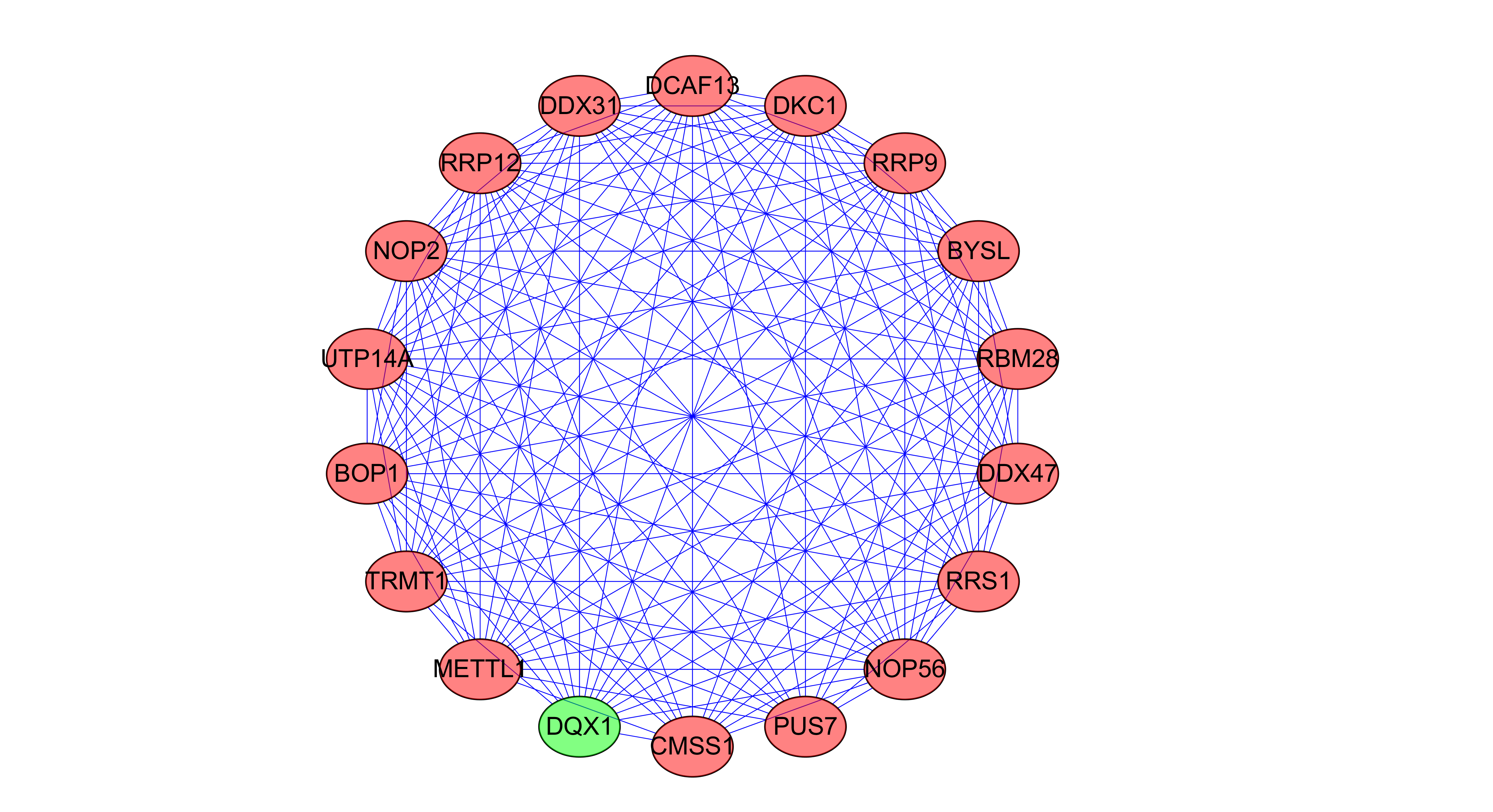

Supplement: Supplemental Information 6 [file peerj-09-11219-s006.zip › raw data/15.cytoscape/subnetwork1.png]

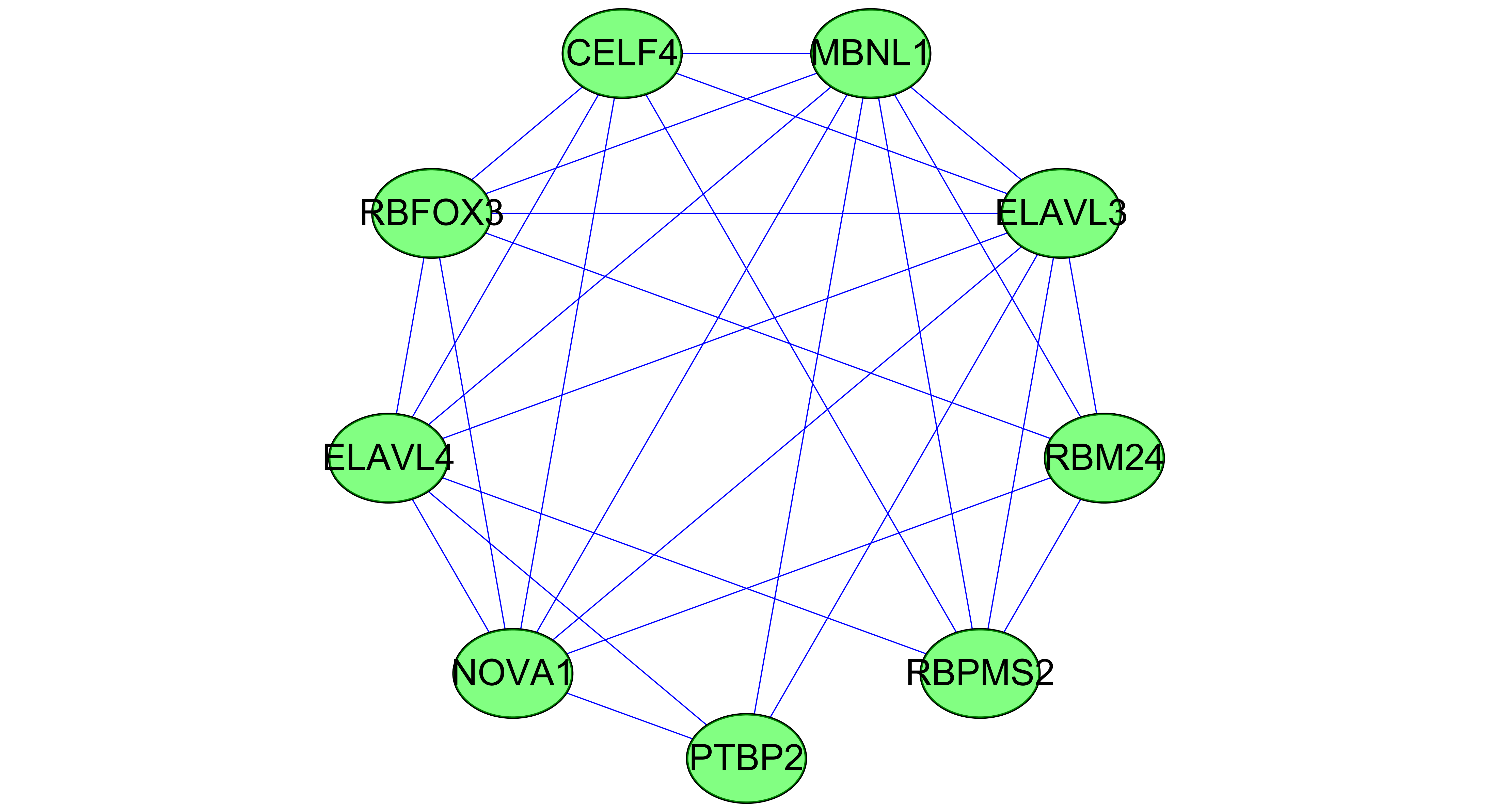

Supplement: Supplemental Information 6 [file peerj-09-11219-s006.zip › raw data/15.cytoscape/subnetwork2.png]

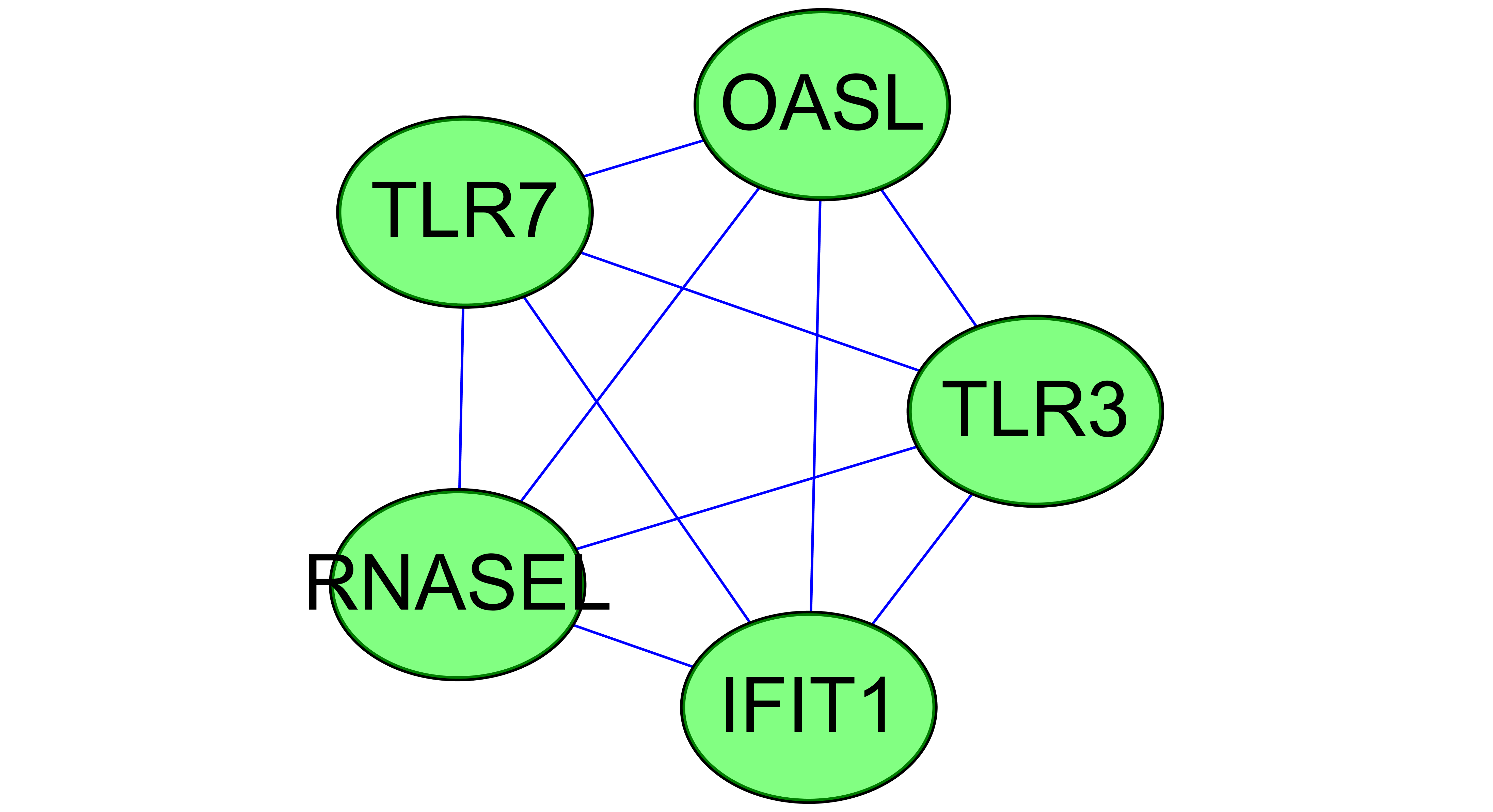

Supplement: Supplemental Information 6 [file peerj-09-11219-s006.zip › raw data/15.cytoscape/subnetwork3.png]

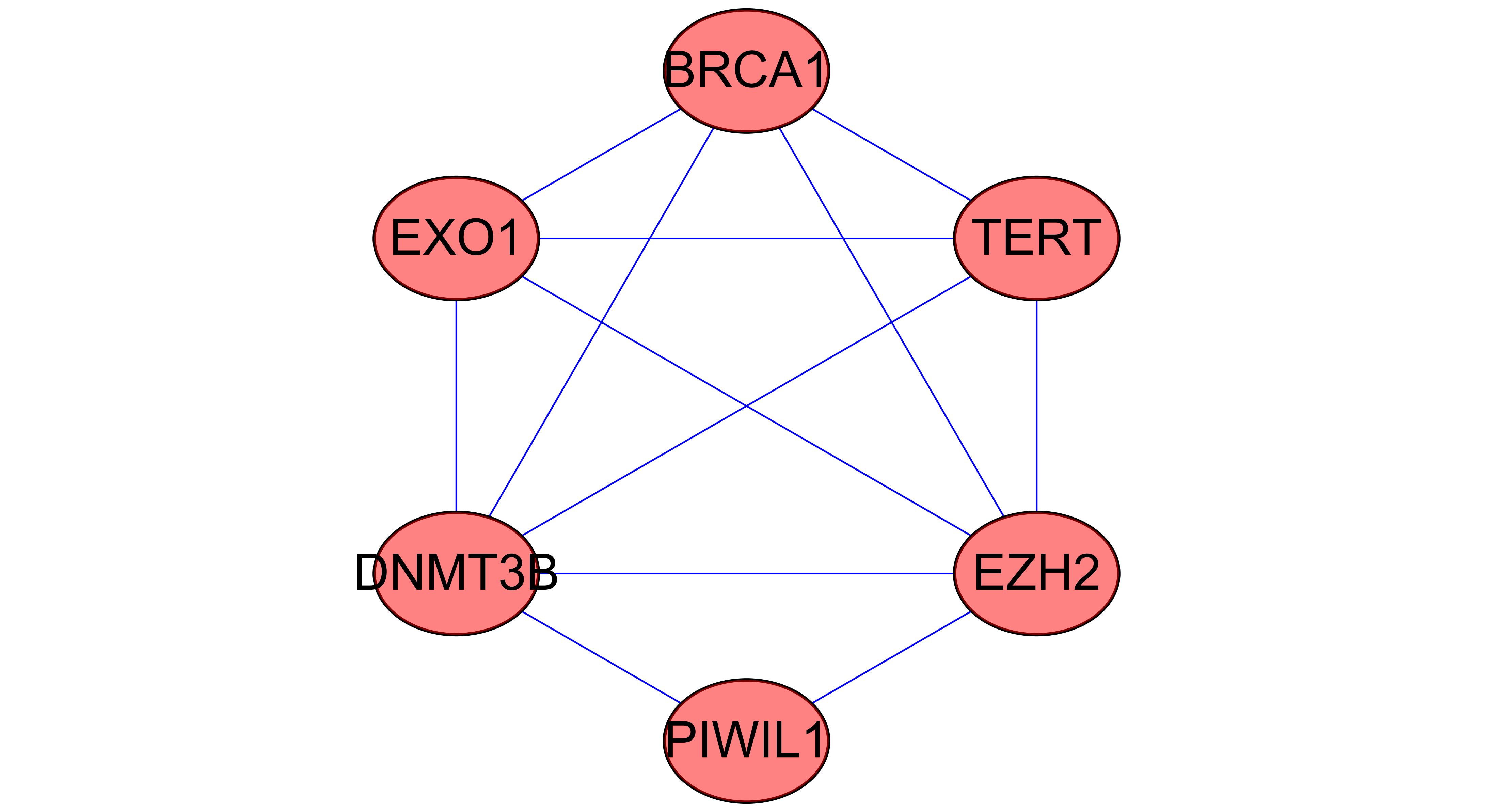

Supplement: Supplemental Information 6 [file peerj-09-11219-s006.zip › raw data/15.cytoscape/subnetwork4.png]

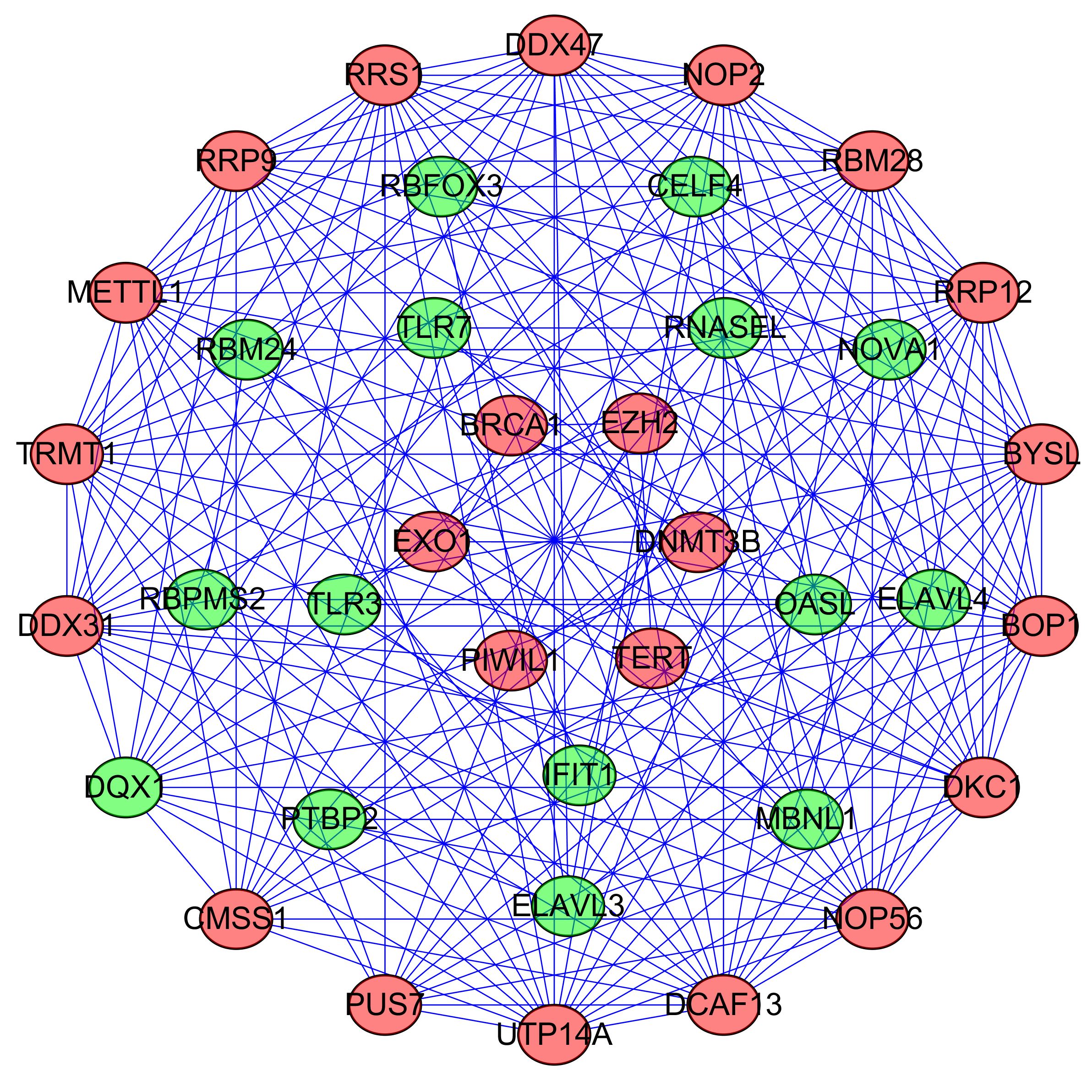

Supplement: Supplemental Information 6 [file peerj-09-11219-s006.zip › raw data/17.subNetwork/subnetwork.png]

|          | pvalue | Hazard ratio       |
|----------|--------|--------------------|
| PNLDC1   | 0.005  | 1.342(1.094–1.647) |
| RRS1     | 0.004  | 1.622(1.171–2.247) |
| HEXIM1   | 0.026  | 1.435(1.045–1.969) |
| PPARGC1A | 0.128  | 0.839(0.669–1.052) |
| PPARGC1B | 0.074  | 0.693(0.464–1.036) |
| BRCA1    | 0.059  | 0.660(0.428–1.016) |
| CELF4    | <0.001 | 1.726(1.307–2.280) |
| AEN      | 0.002  | 1.549(1.169–2.052) |
| NOVA1    | 0.121  | 1.178(0.958–1.449) |

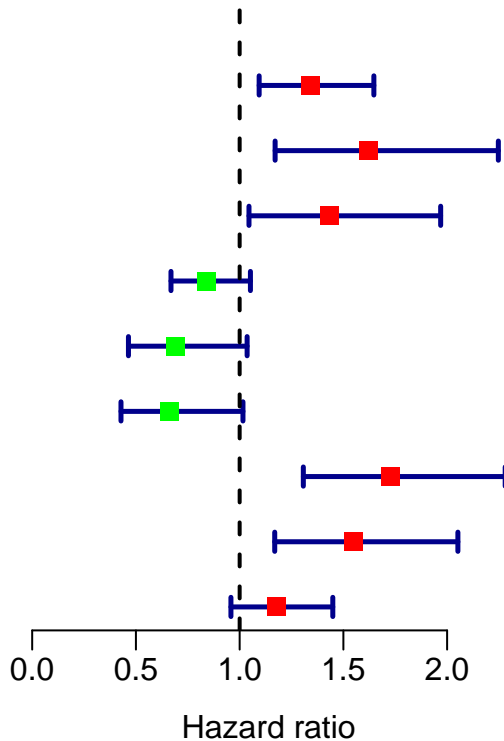

Supplement: Supplemental Information 6 [file peerj-09-11219-s006.zip › raw data/22.tcga-geoModel/multiCox.pdf]

Risk + High risk + Low risk

Survival probability

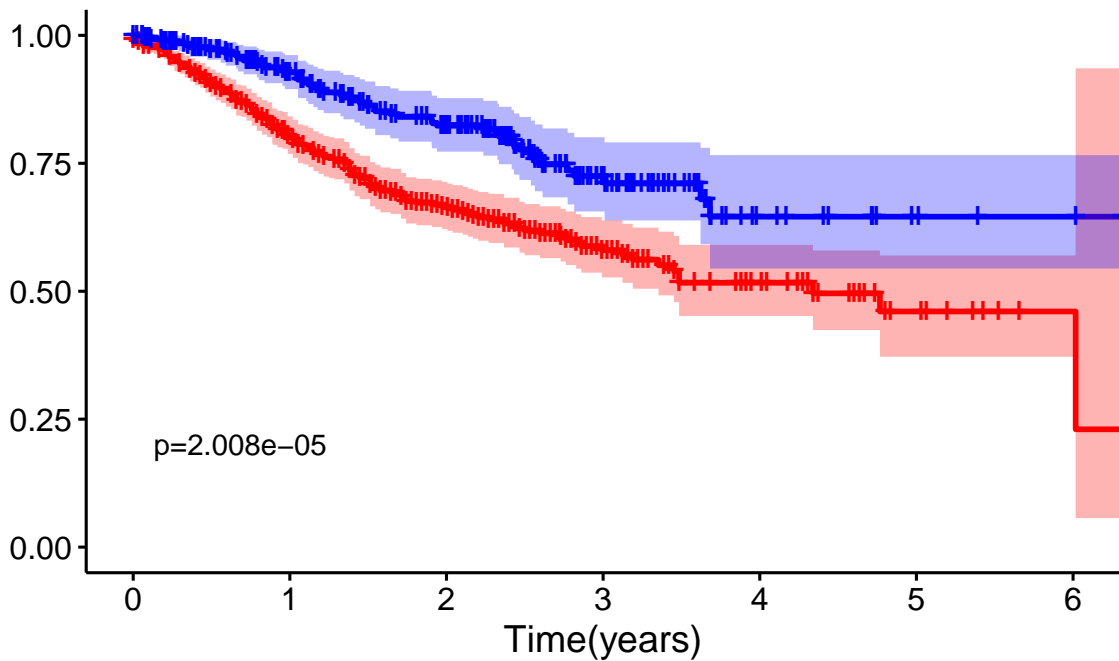

$p=2.008e-05$

| Risk      | Time(years) |     |     |    |    |    |   |
|-----------|-------------|-----|-----|----|----|----|---|
|           | 0           | 1   | 2   | 3  | 4  | 5  | 6 |
| High risk | 483         | 337 | 193 | 71 | 34 | 10 | 2 |
| Low risk  | 275         | 194 | 138 | 52 | 12 | 4  | 2 |

Supplement: Supplemental Information 6 [file peerj-09-11219-s006.zip › raw data/24.survival/TCGA-GEOmodel/testSurv.pdf]

Risk + High risk + Low risk

Survival probability

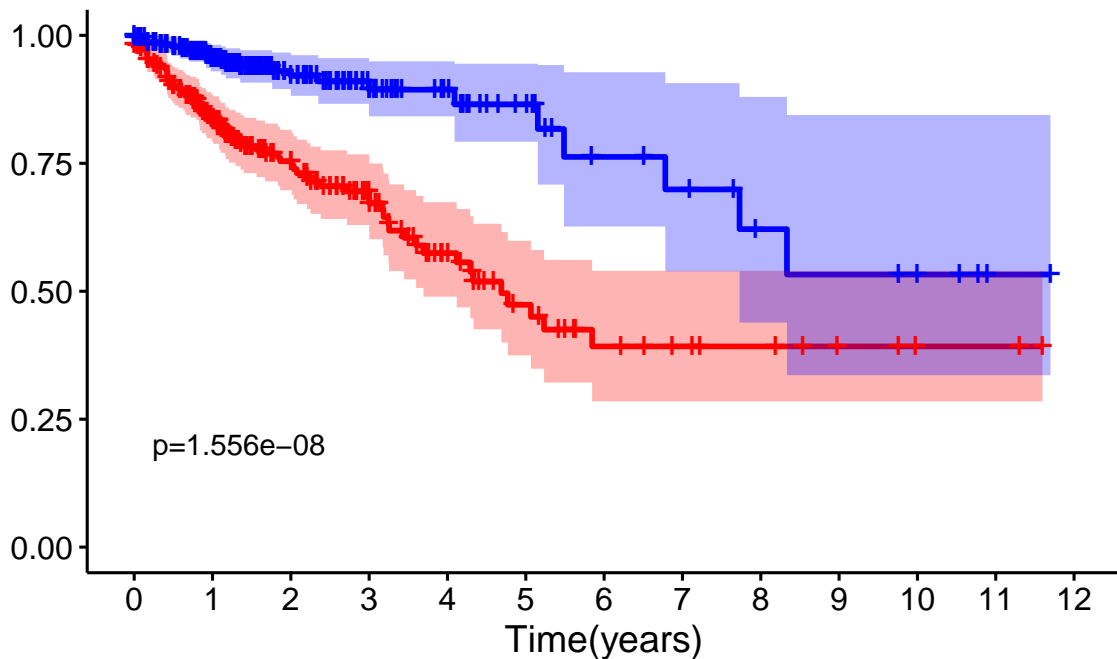

$p=1.556e-08$

Risk High risk  
Low risk

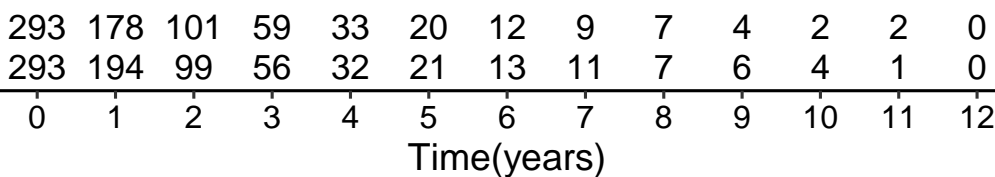

Supplement: Supplemental Information 6 [file peerj-09-11219-s006.zip › raw data/24.survival/TCGA-GEOmodel/trainSurv.pdf]

**ROC curve ( AUC = 0.638 )**

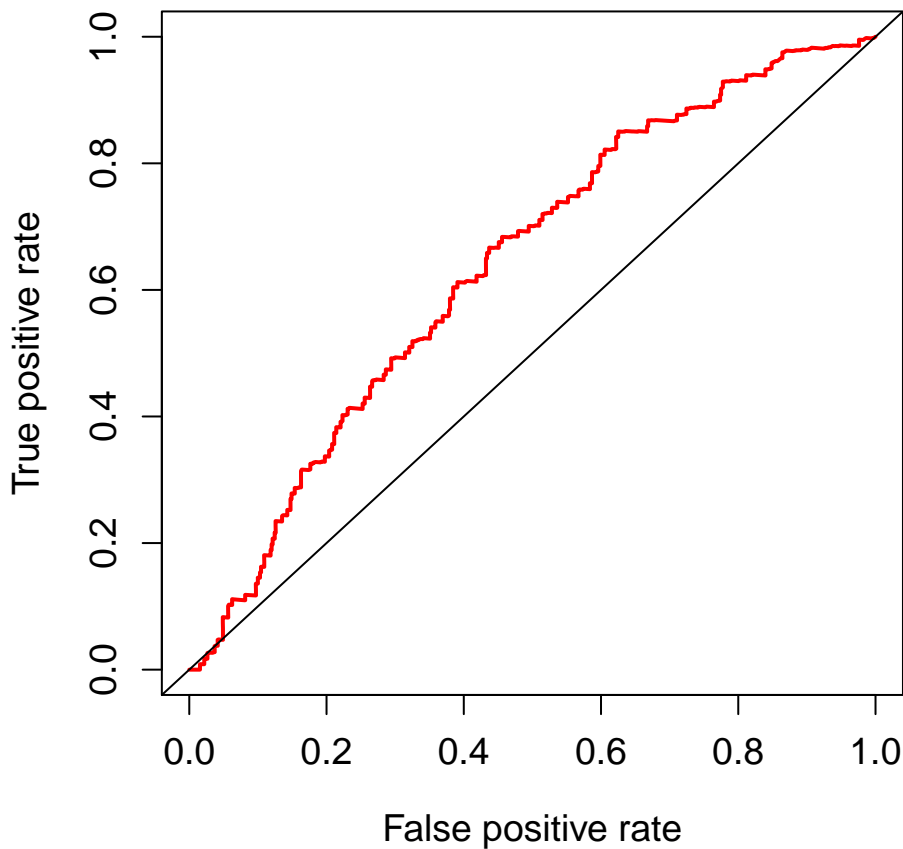

Supplement: Supplemental Information 6 [file peerj-09-11219-s006.zip › raw data/25.ROC/TCGA-GEO model/testROC.pdf]

**ROC curve ( AUC = 0.712 )**

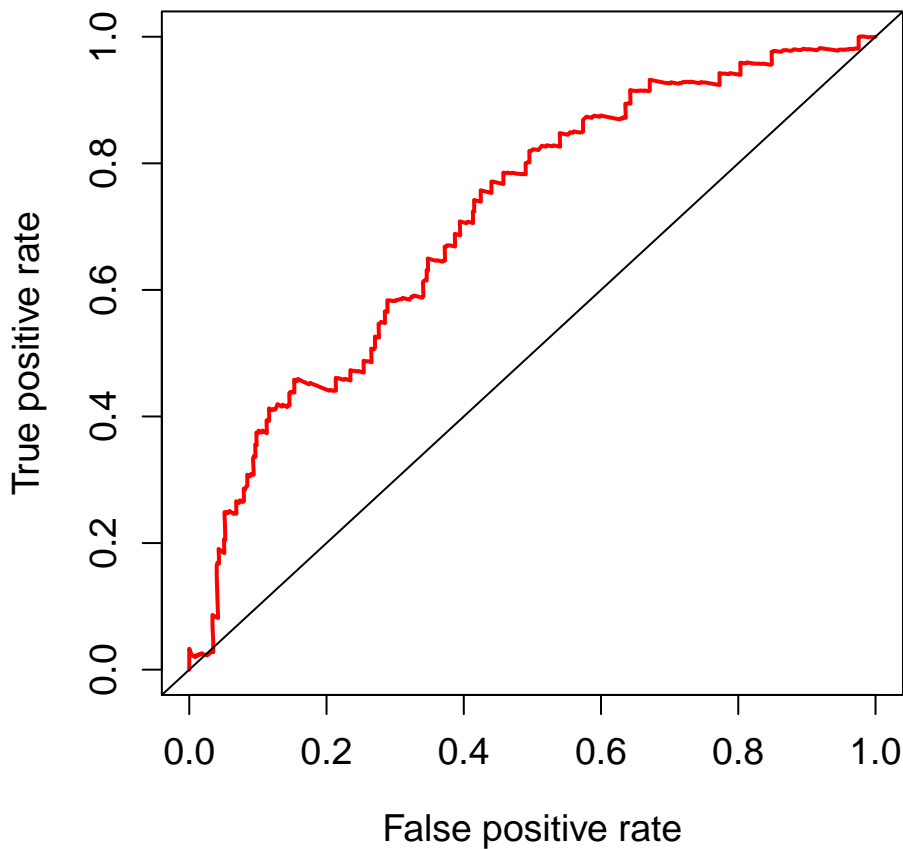

Supplement: Supplemental Information 6 [file peerj-09-11219-s006.zip › raw data/25.ROC/TCGA-GEO model/trainROC.pdf]

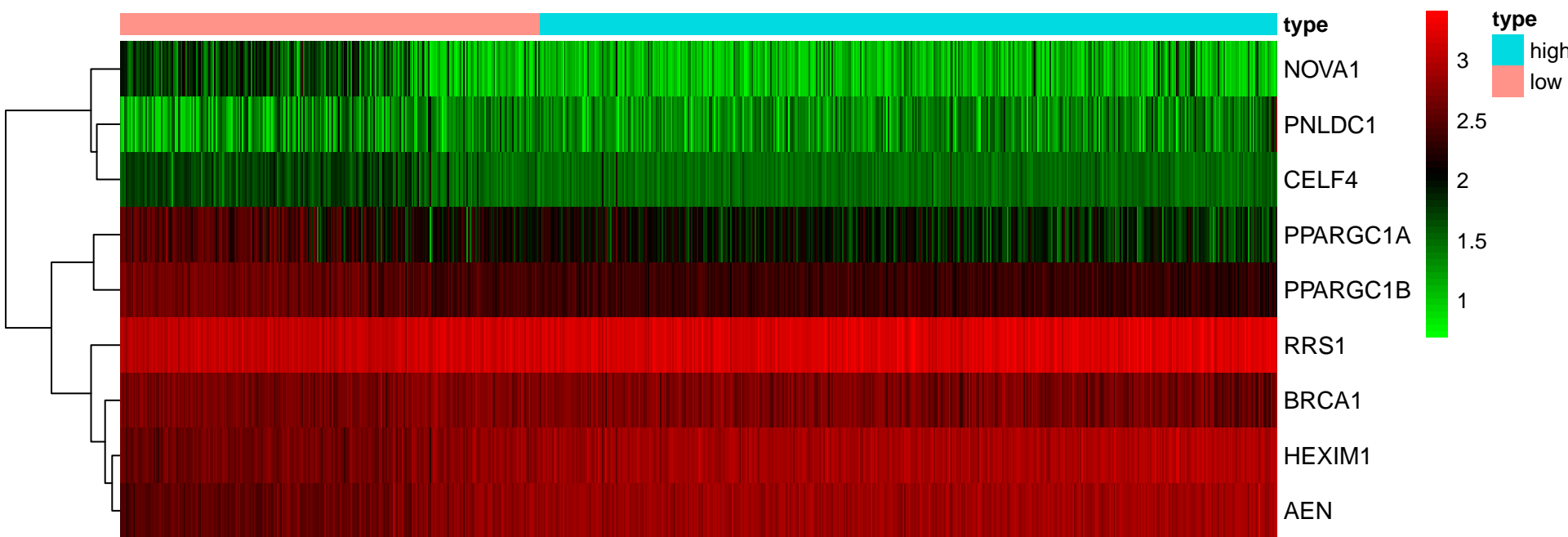

Supplement: Supplemental Information 6 [file peerj-09-11219-s006.zip › raw data/26.riskPlot/TCGA-GEO model/test.heatmap.pdf]

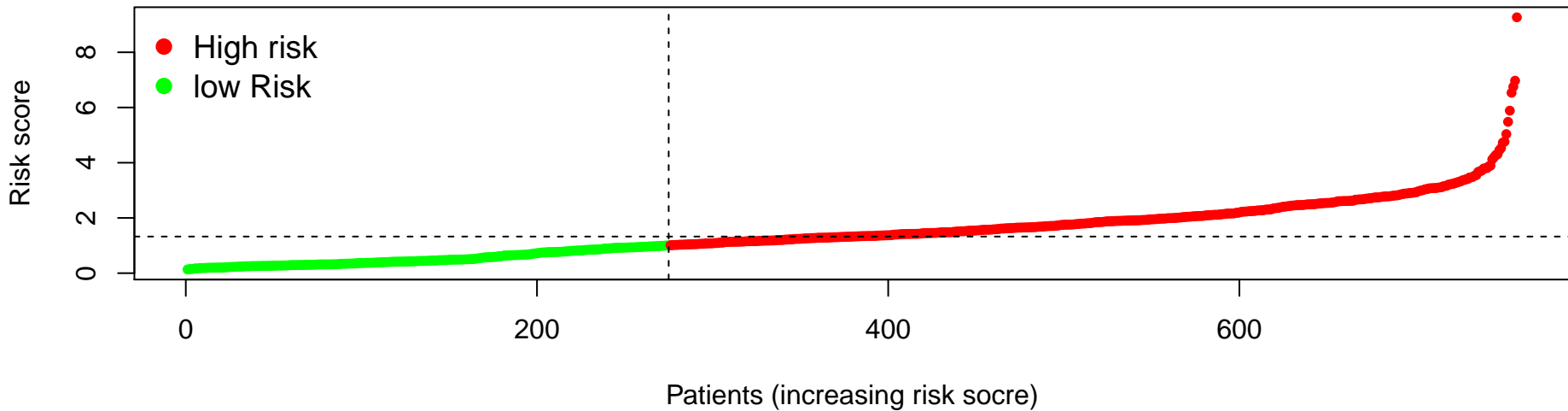

Supplement: Supplemental Information 6 [file peerj-09-11219-s006.zip › raw data/26.riskPlot/TCGA-GEO model/test.riskScore.pdf]

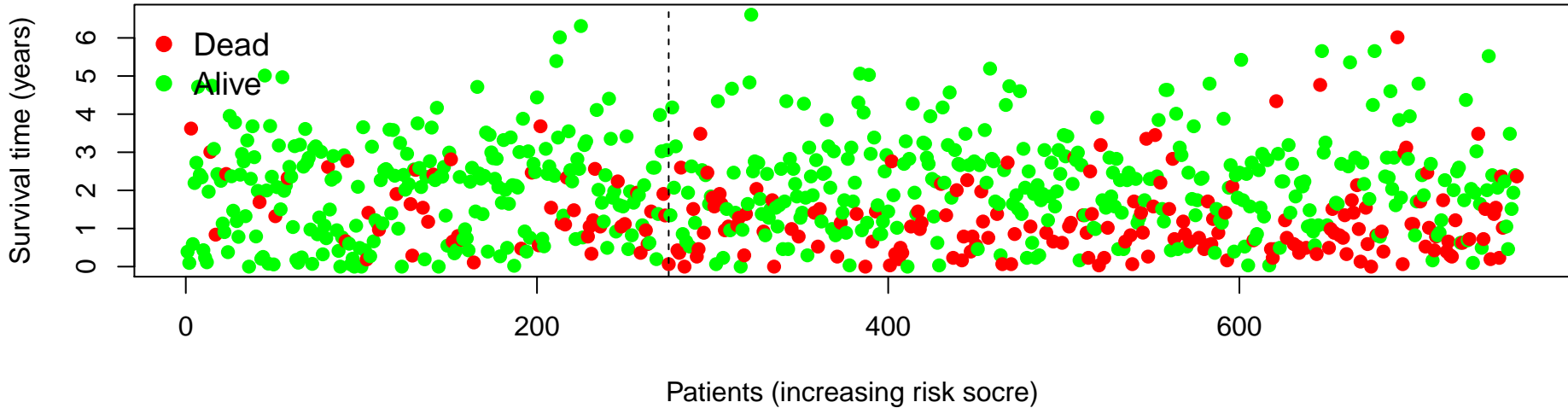

Supplement: Supplemental Information 6 [file peerj-09-11219-s006.zip › raw data/26.riskPlot/TCGA-GEO model/test.survStat.pdf]

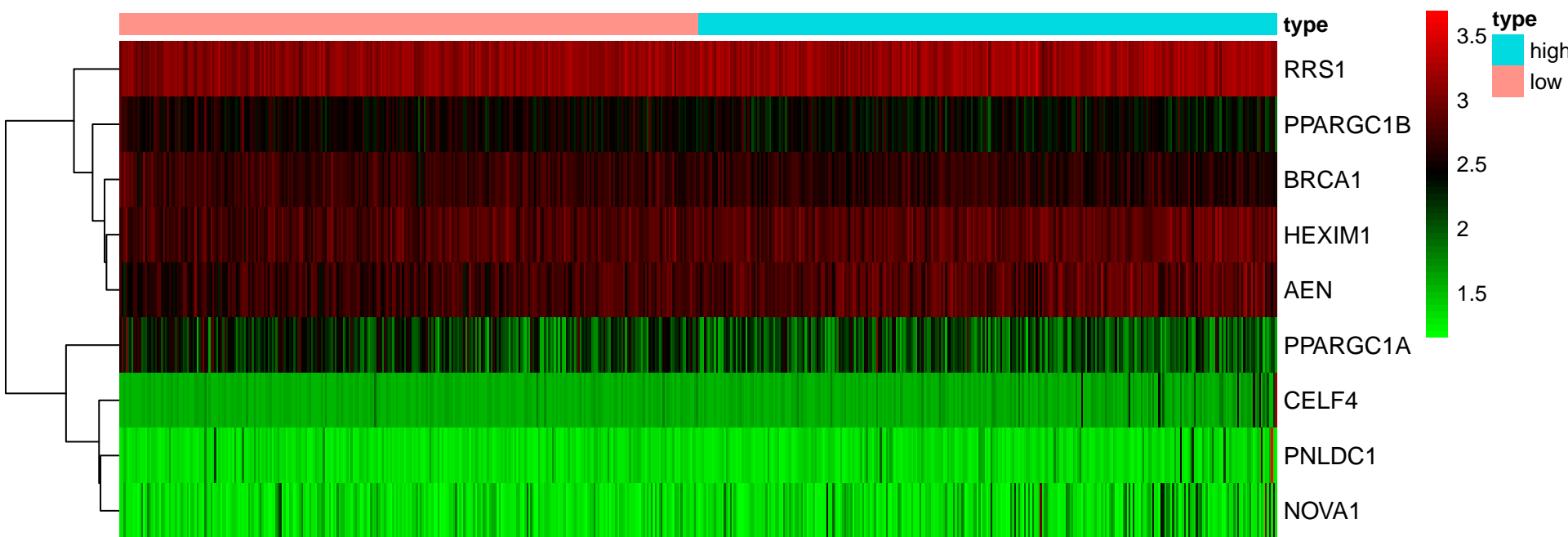

Supplement: Supplemental Information 6 [file peerj-09-11219-s006.zip › raw data/26.riskPlot/TCGA-GEO model/train.heatmap.pdf]

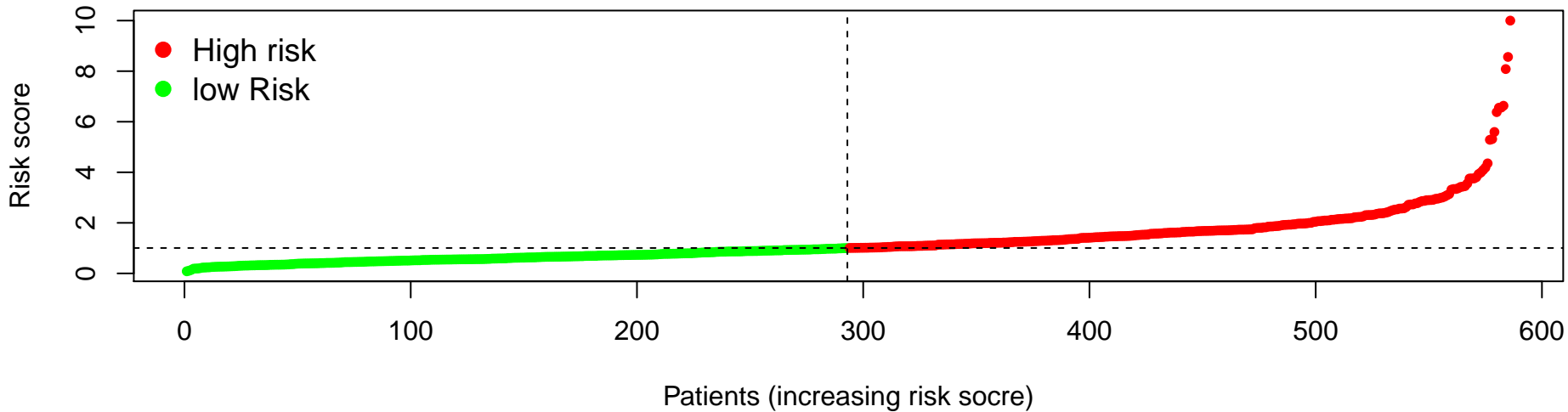

Supplement: Supplemental Information 6 [file peerj-09-11219-s006.zip › raw data/26.riskPlot/TCGA-GEO model/train.riskScore.pdf]

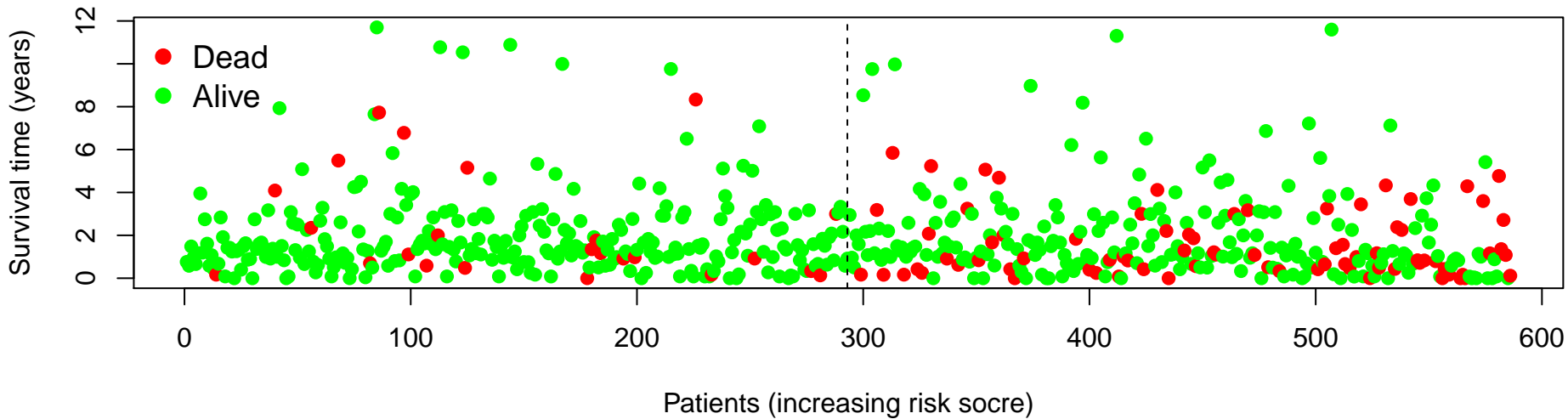

Supplement: Supplemental Information 6 [file peerj-09-11219-s006.zip › raw data/26.riskPlot/TCGA-GEO model/train.survStat.pdf]

|           | pvalue | Hazard ratio       |
|-----------|--------|--------------------|
| age       | <0.001 | 1.053(1.032–1.075) |
| gender    | 0.462  | 0.855(0.562–1.299) |
| stage     | 0.099  | 1.765(0.899–3.464) |
| T         | 0.049  | 1.607(1.002–2.577) |
| M         | 0.341  | 1.575(0.618–4.012) |
| N         | 0.501  | 1.152(0.762–1.742) |
| riskScore | <0.001 | 1.083(1.050–1.117) |

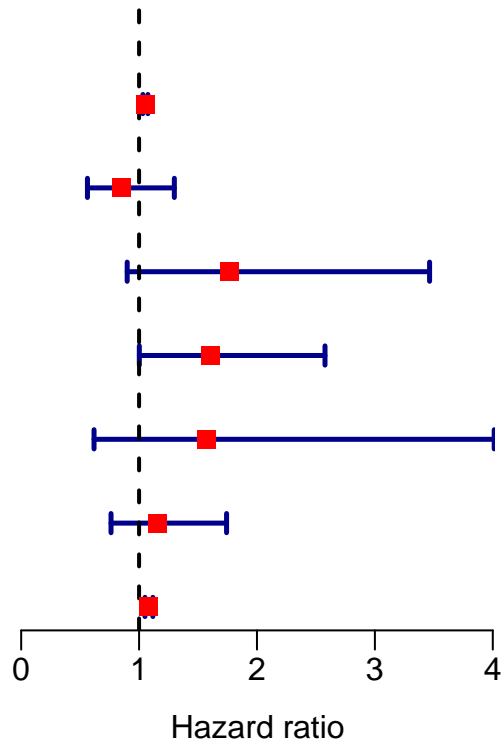

Supplement: Supplemental Information 6 [file peerj-09-11219-s006.zip › raw data/27.trainIndep/train.multiForest.pdf]

|           | pvalue | Hazard ratio       |
|-----------|--------|--------------------|
| age       | <0.001 | 1.036(1.016–1.057) |
| gender    | 0.719  | 1.078(0.715–1.625) |
| stage     | <0.001 | 2.438(1.923–3.090) |
| T         | <0.001 | 2.998(1.988–4.521) |
| M         | <0.001 | 4.865(3.198–7.401) |
| N         | <0.001 | 2.079(1.638–2.640) |
| riskScore | <0.001 | 1.084(1.057–1.113) |

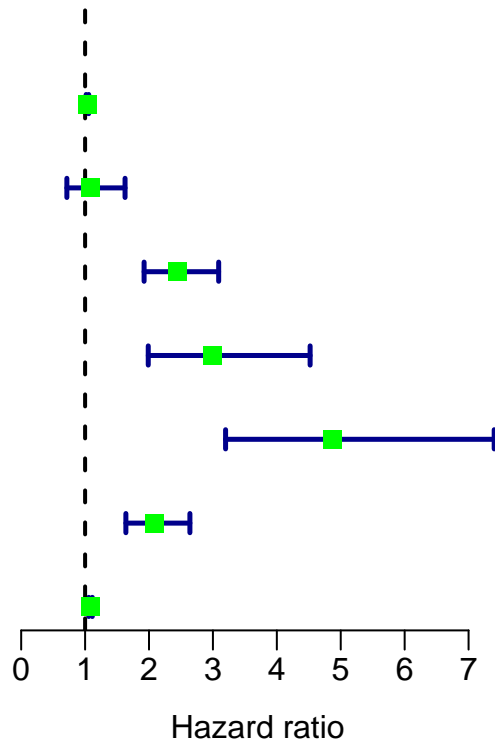

Supplement: Supplemental Information 6 [file peerj-09-11219-s006.zip › raw data/27.trainIndep/train.uniForest.pdf]

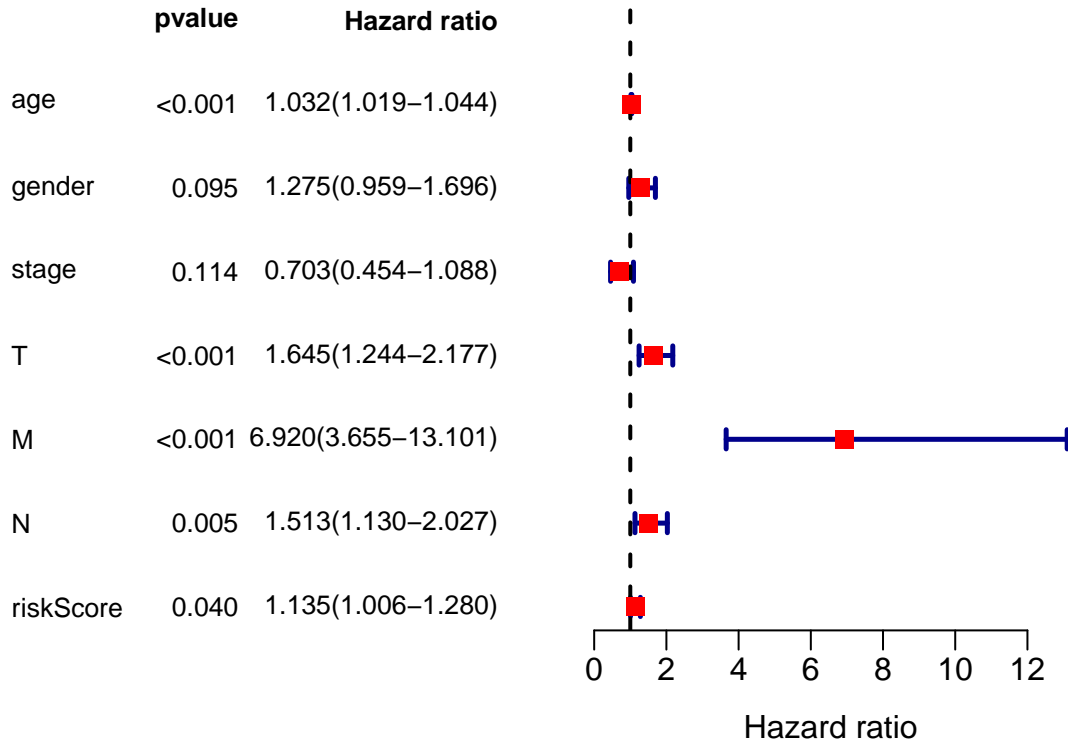

Supplement: Supplemental Information 6 [file peerj-09-11219-s006.zip › raw data/28.testIndep/test.multiForest.pdf]

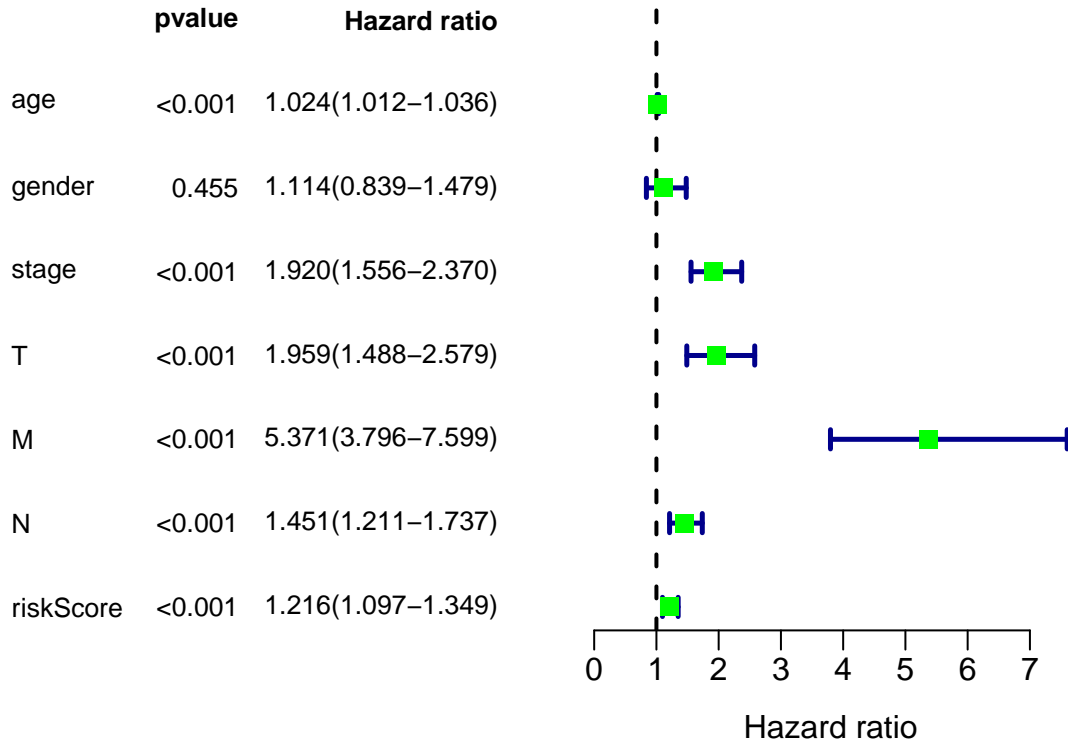

Supplement: Supplemental Information 6 [file peerj-09-11219-s006.zip › raw data/28.testIndep/test.uniForest.pdf]

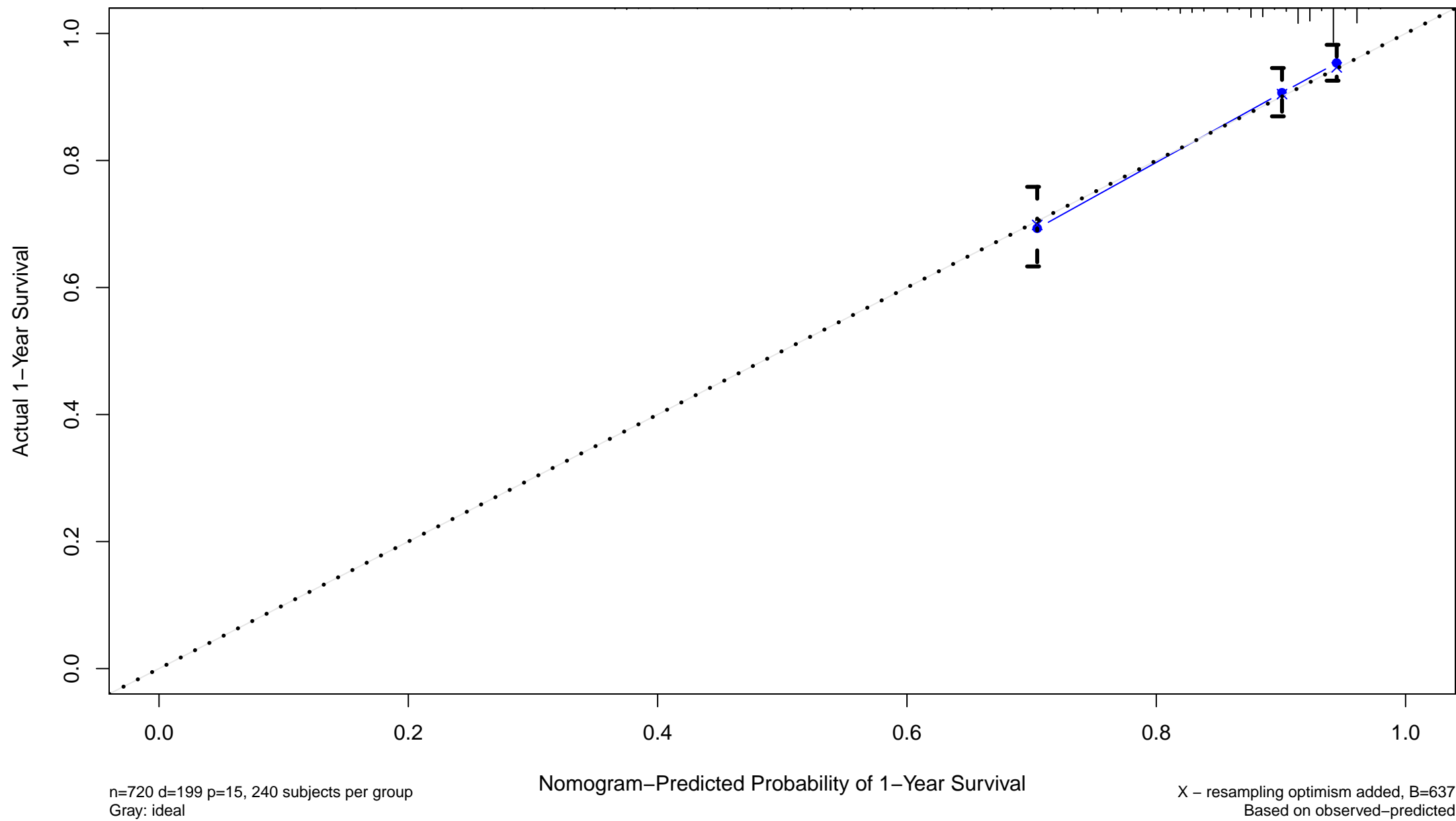

Supplement: Supplemental Information 6 [file peerj-09-11219-s006.zip › raw data/29.nomogram/20.C-index/GEO/5_Calibration/3points/calibrate1.pdf]

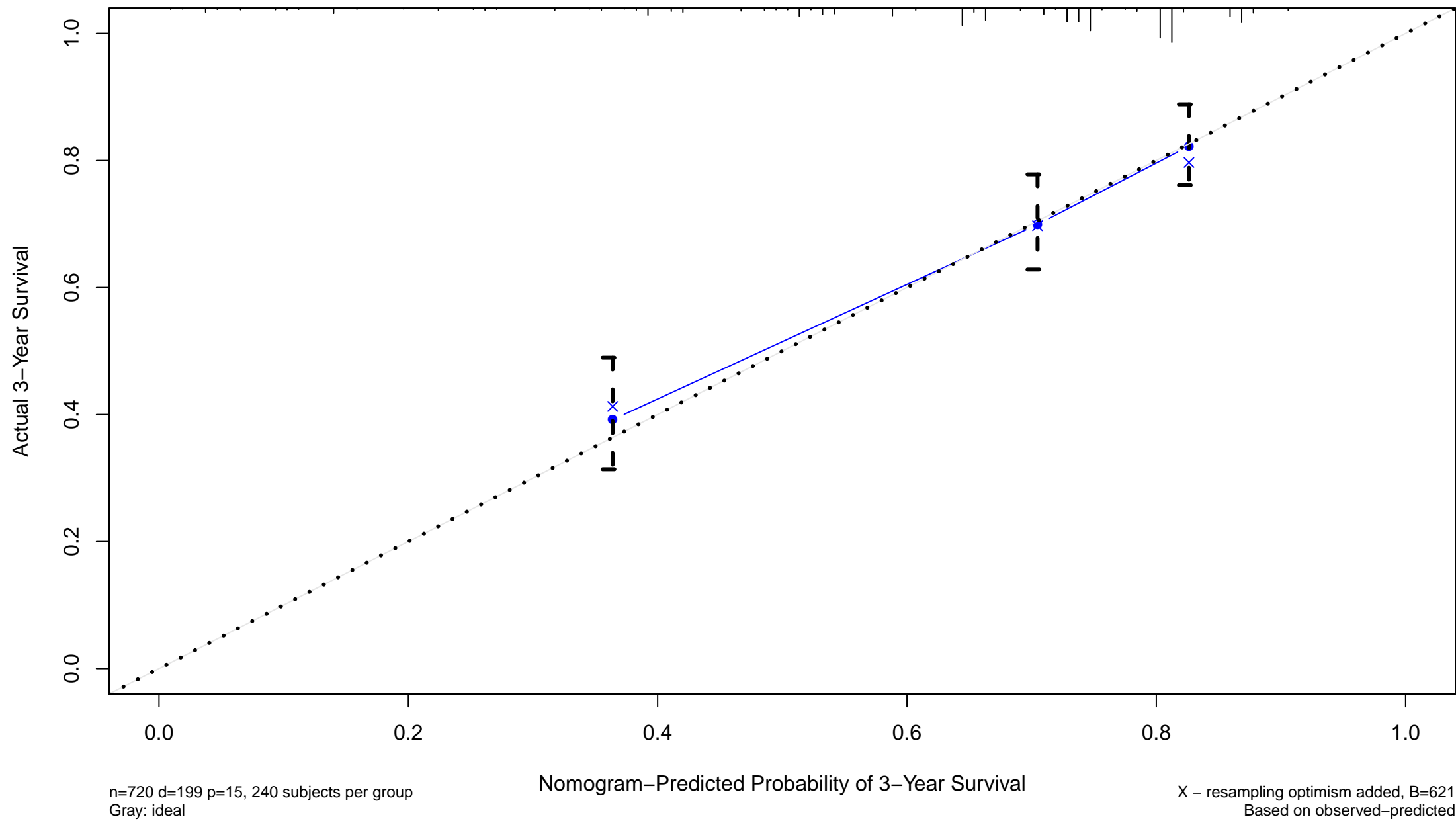

Supplement: Supplemental Information 6 [file peerj-09-11219-s006.zip › raw data/29.nomogram/20.C-index/GEO/5_Calibration/3points/calibrate3.pdf]

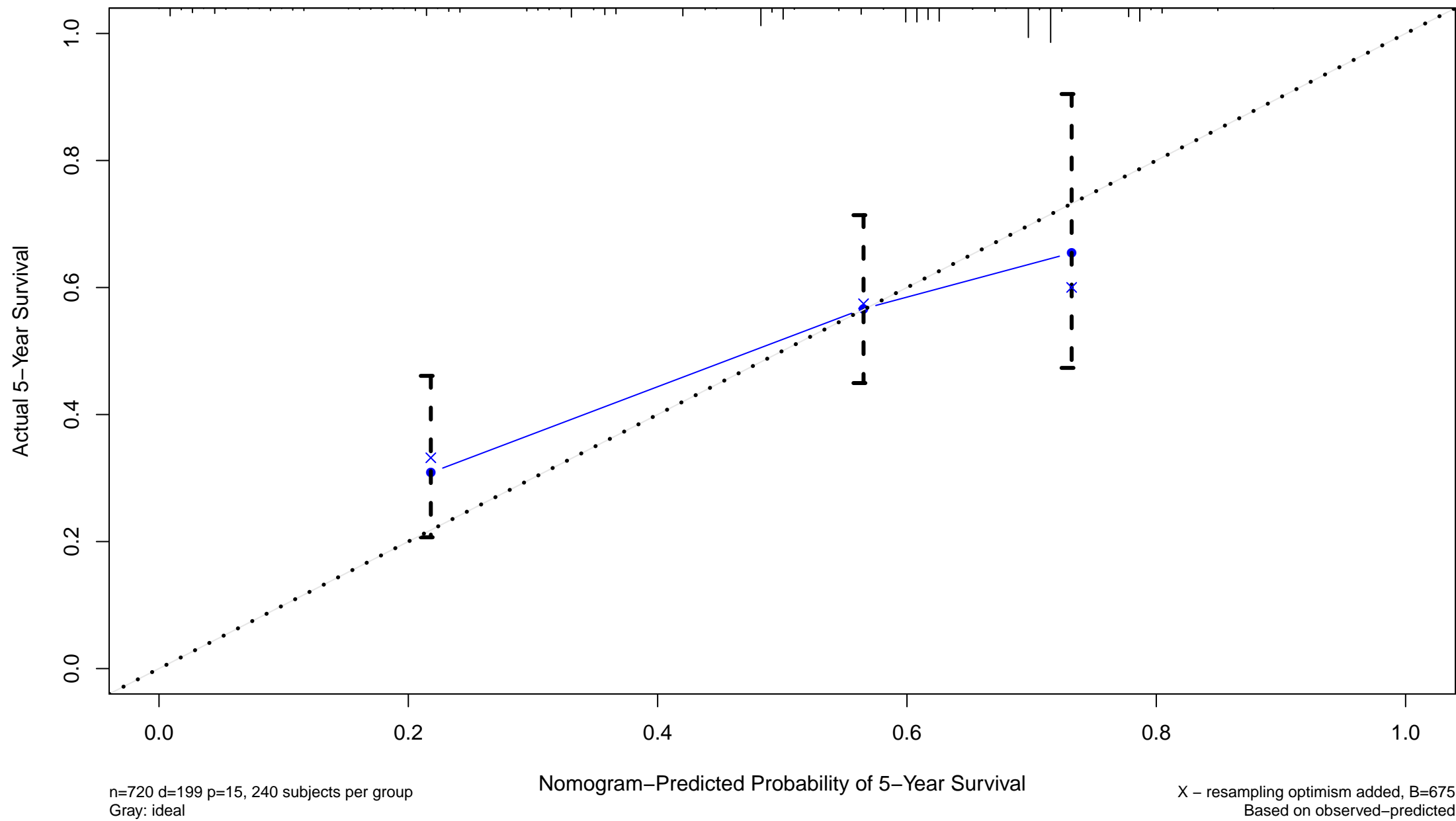

Supplement: Supplemental Information 6 [file peerj-09-11219-s006.zip › raw data/29.nomogram/20.C-index/GEO/5_Calibration/3points/calibrate5.pdf]

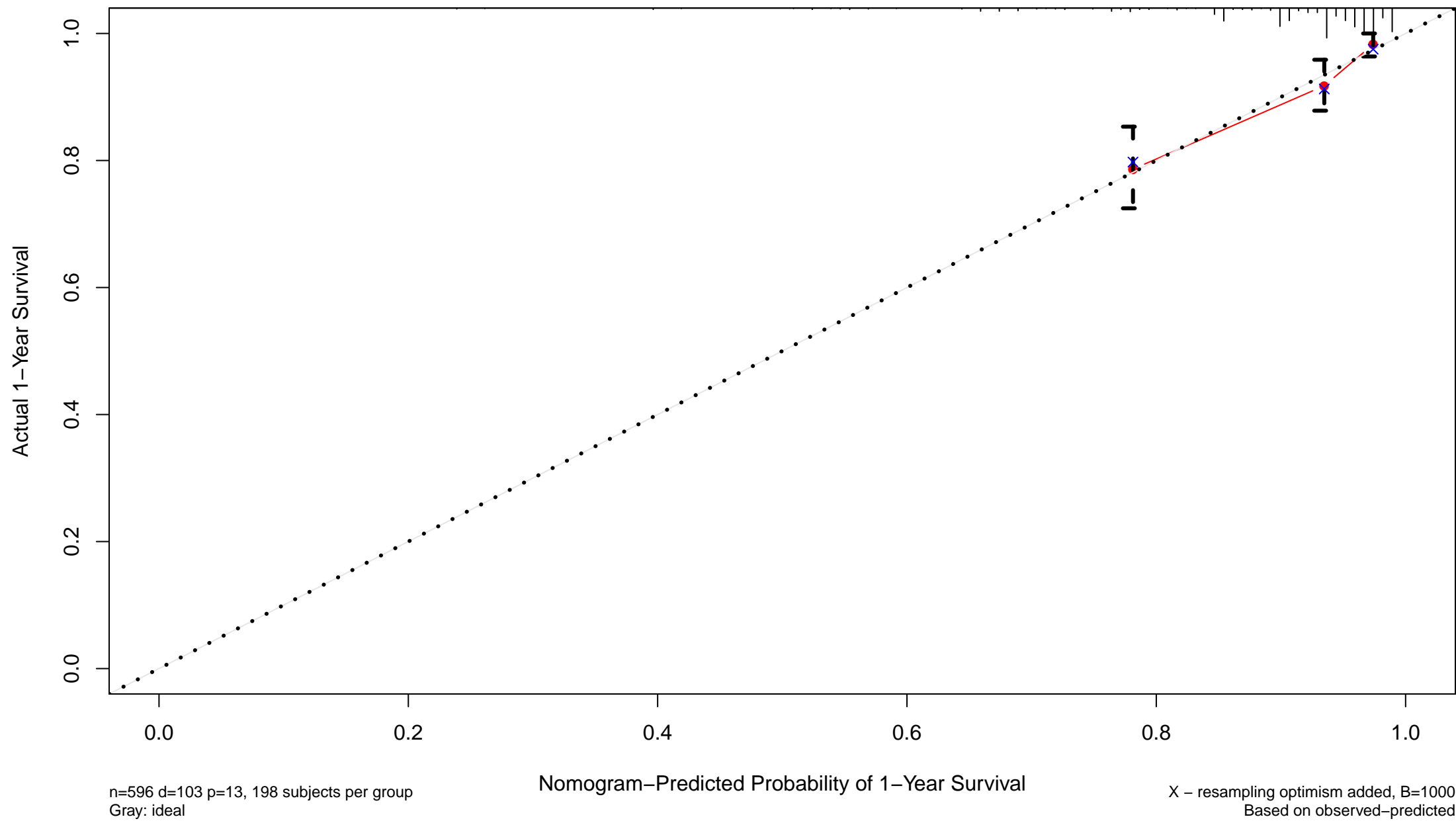

Supplement: Supplemental Information 6 [file peerj-09-11219-s006.zip › raw data/29.nomogram/20.C-index/TCGA/5_Calibration/3points/calibrate1.pdf]

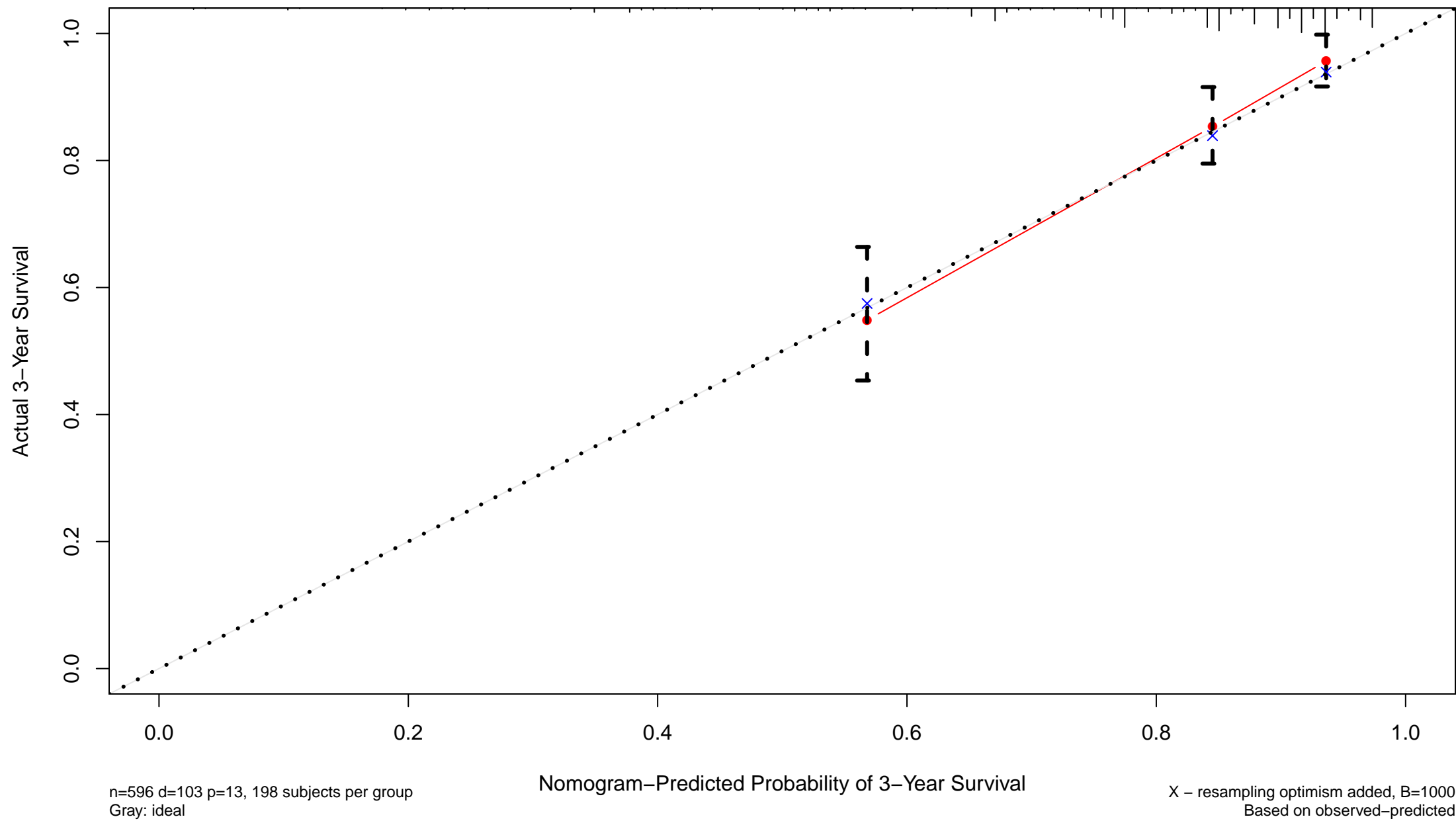

Supplement: Supplemental Information 6 [file peerj-09-11219-s006.zip › raw data/29.nomogram/20.C-index/TCGA/5_Calibration/3points/calibrate3.pdf]

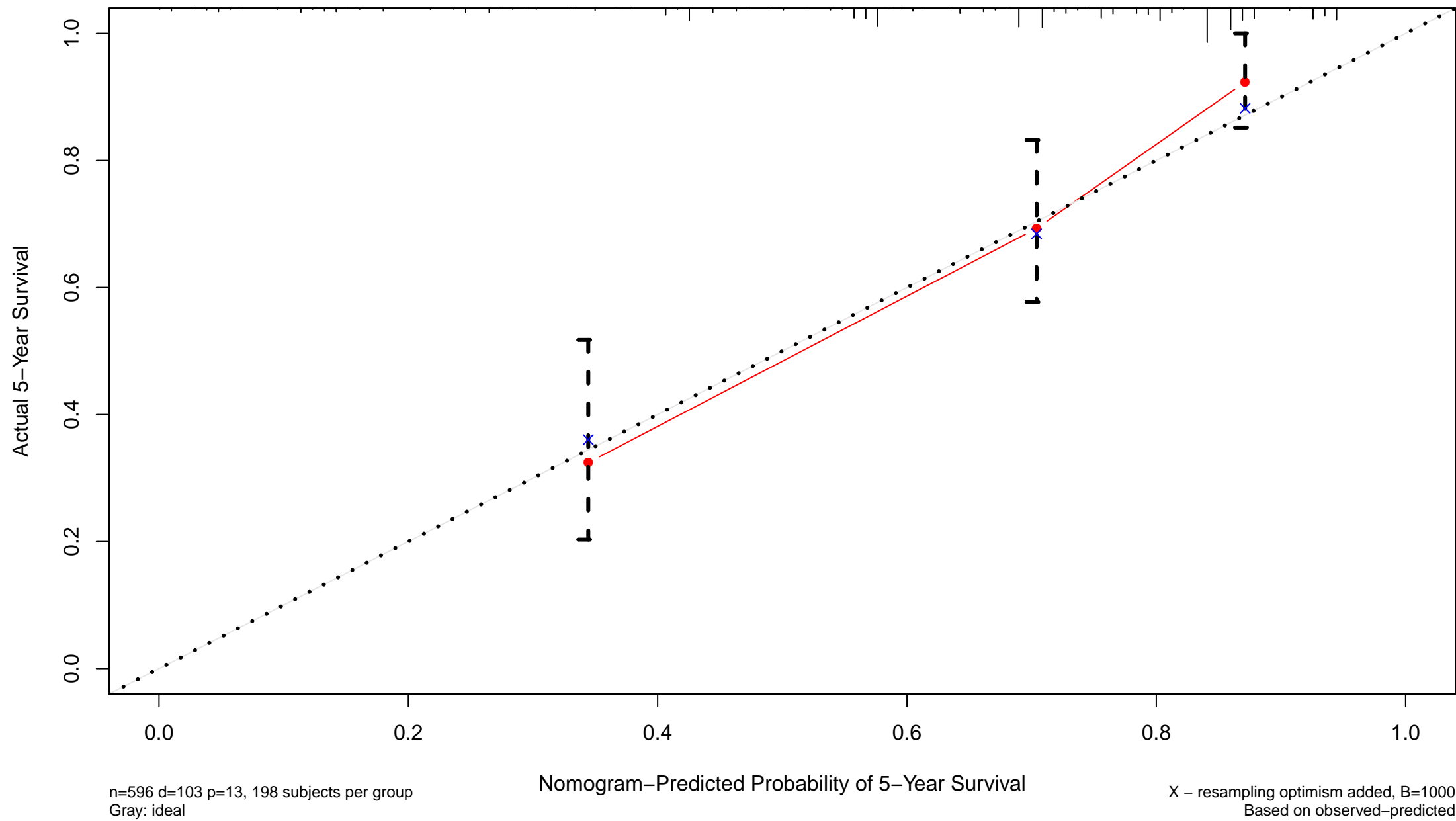

Supplement: Supplemental Information 6 [file peerj-09-11219-s006.zip › raw data/29.nomogram/20.C-index/TCGA/5_Calibration/3points/calibrate5.pdf]

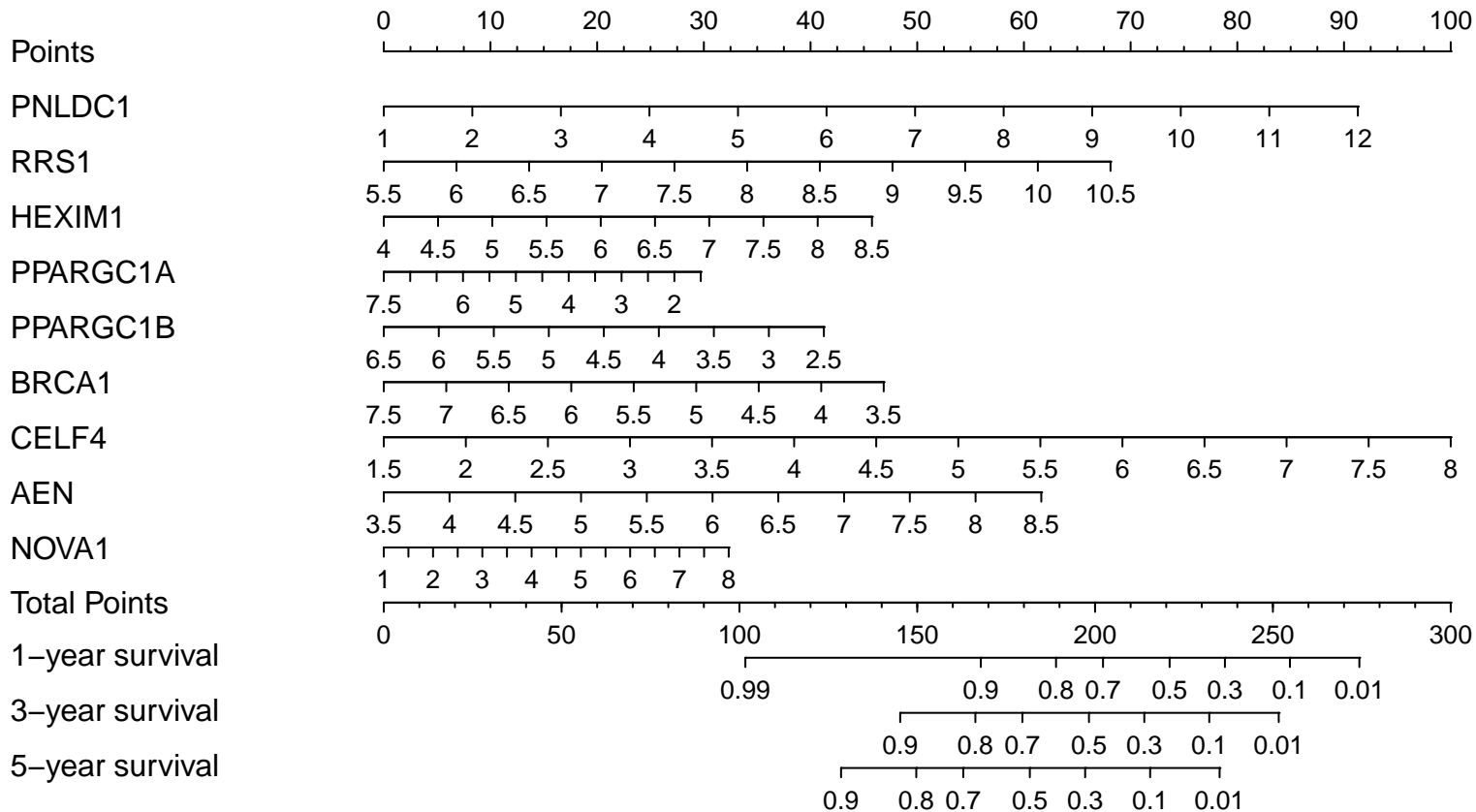

Supplement: Supplemental Information 6 [file peerj-09-11219-s006.zip › raw data/29.nomogram/train.Nomogram.pdf]

**Cor=0.642 (p=9.433e-82)**

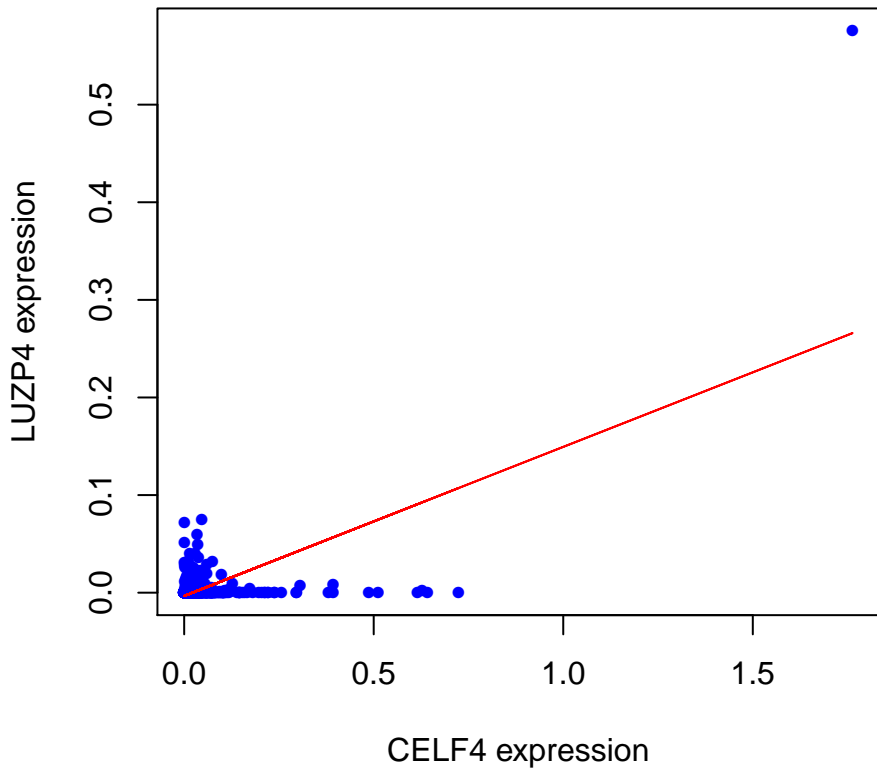

Supplement: Supplemental Information 6 [file peerj-09-11219-s006.zip › raw data/31.conexpression/17.cor/cor.CELF4_LUZP4.pdf]

**Cor=0.771 (p=2.784e-137)**

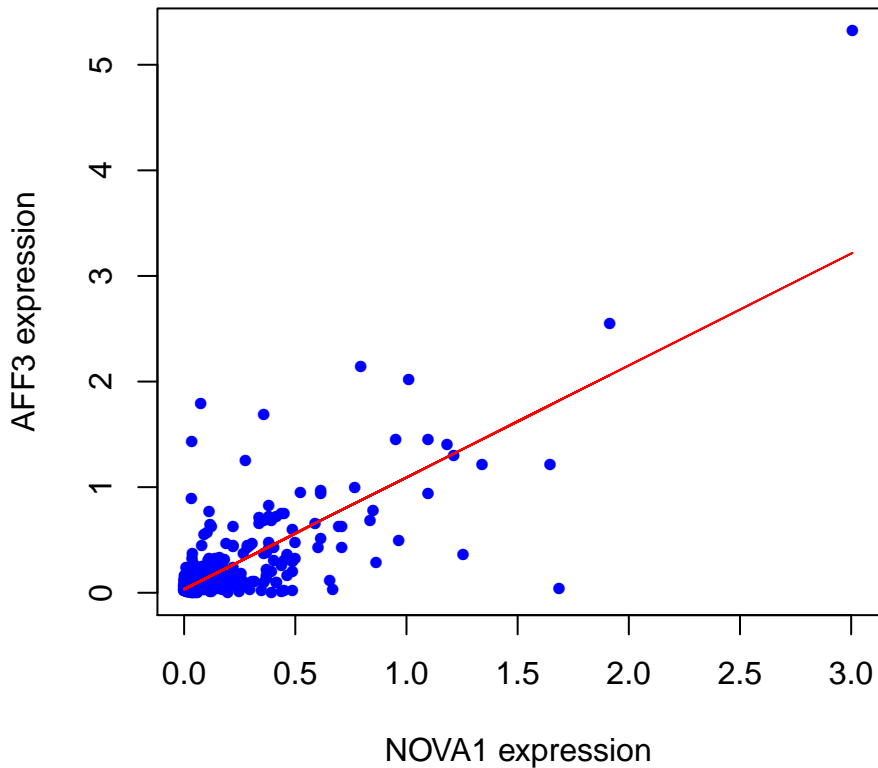

Supplement: Supplemental Information 6 [file peerj-09-11219-s006.zip › raw data/31.conexpression/17.cor/cor.NOVA1_AFF3.pdf]

**Cor=0.839 (p=1.696e-184)**

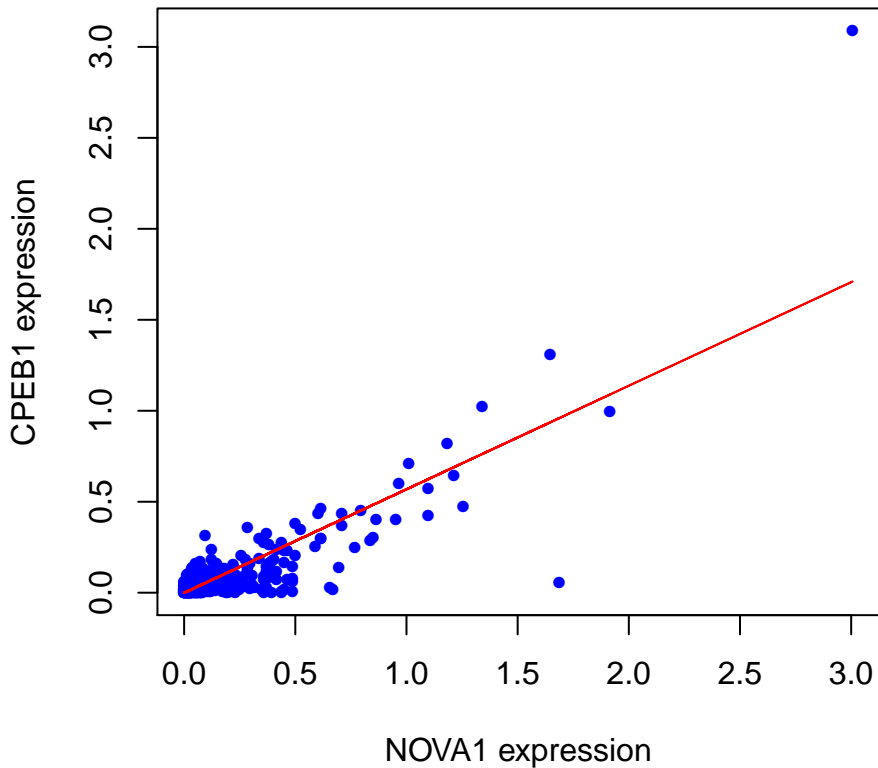

Supplement: Supplemental Information 6 [file peerj-09-11219-s006.zip › raw data/31.conexpression/17.cor/cor.NOVA1_CPEB1.pdf]

**Cor=0.709 (p=1.229e-106)**

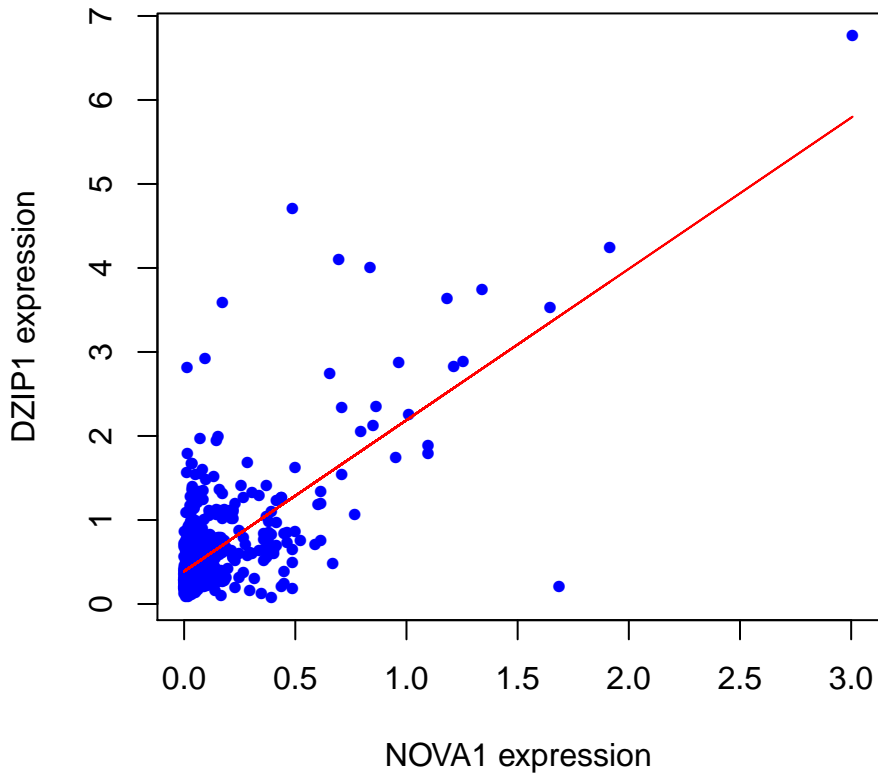

Supplement: Supplemental Information 6 [file peerj-09-11219-s006.zip › raw data/31.conexpression/17.cor/cor.NOVA1_DZIP1.pdf]

**Cor=0.721 (p=7.254e-112)**

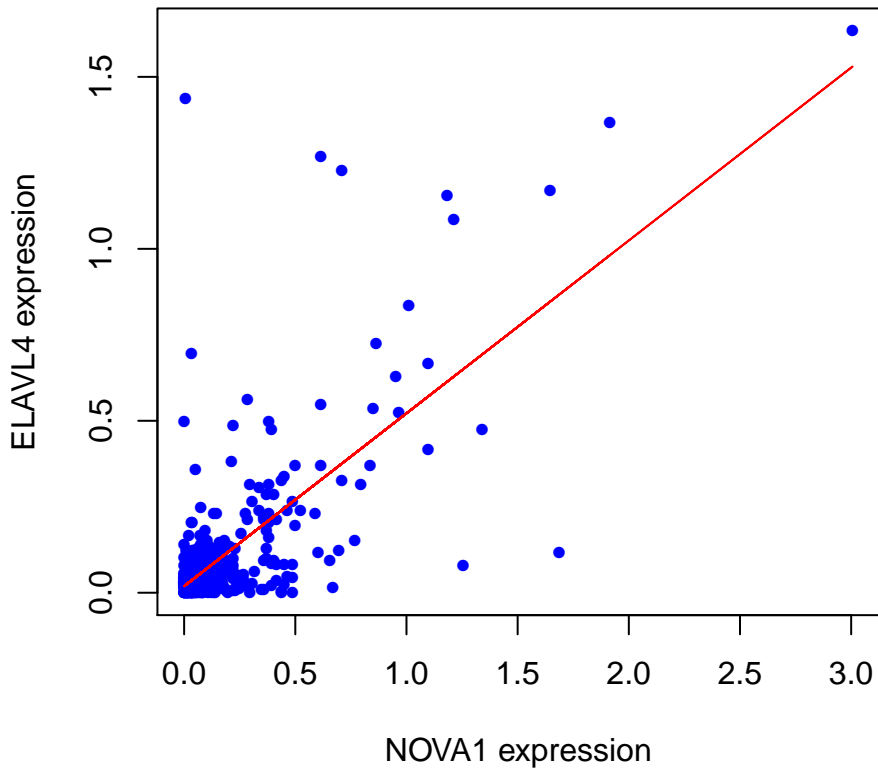

Supplement: Supplemental Information 6 [file peerj-09-11219-s006.zip › raw data/31.conexpression/17.cor/cor.NOVA1_ELAVL4.pdf]

**Cor=0.74 ( $p=5.511\text{e-}121$ )**

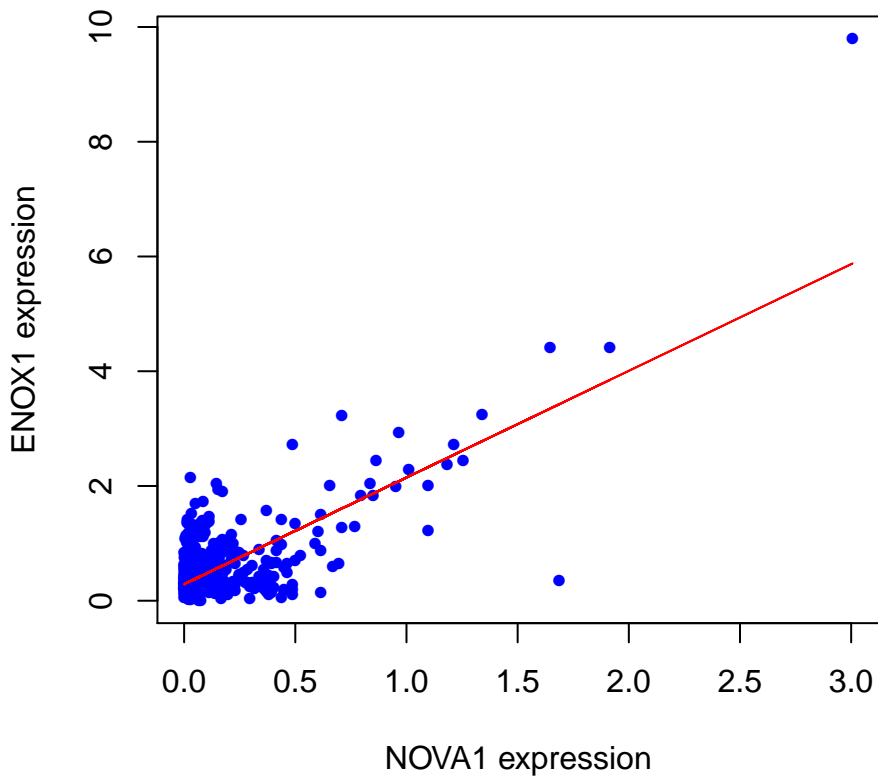

Supplement: Supplemental Information 6 [file peerj-09-11219-s006.zip › raw data/31.conexpression/17.cor/cor.NOVA1_ENOX1.pdf]

**Cor=0.68 ( $p=2.954e-95$ )**

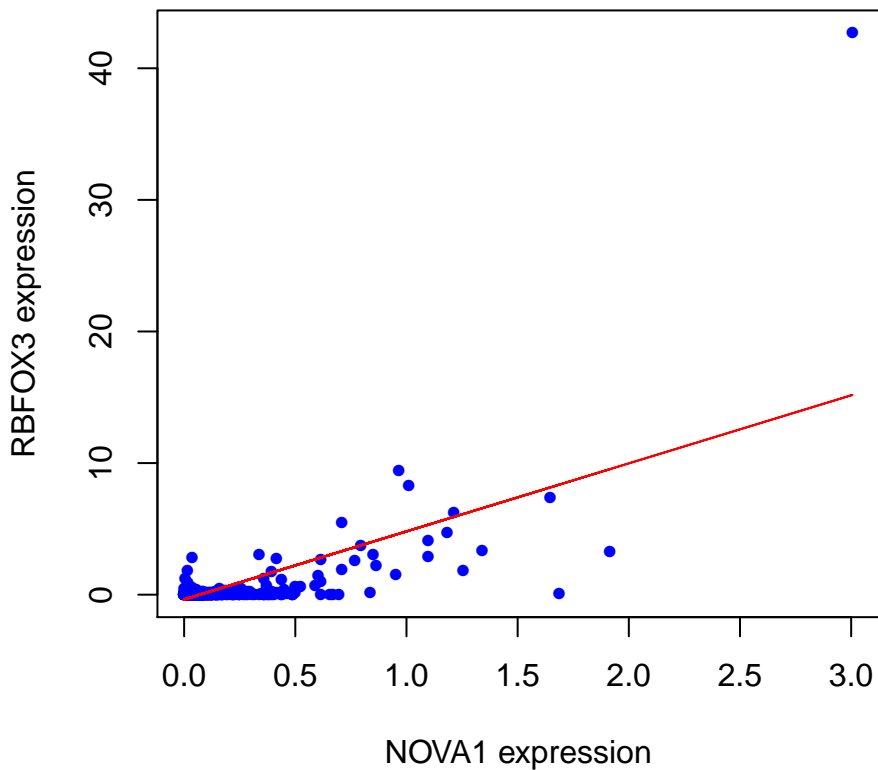

Supplement: Supplemental Information 6 [file peerj-09-11219-s006.zip › raw data/31.conexpression/17.cor/cor.NOVA1_RBFOX3.pdf]

**Cor=0.611 (p=4.333e-72)**

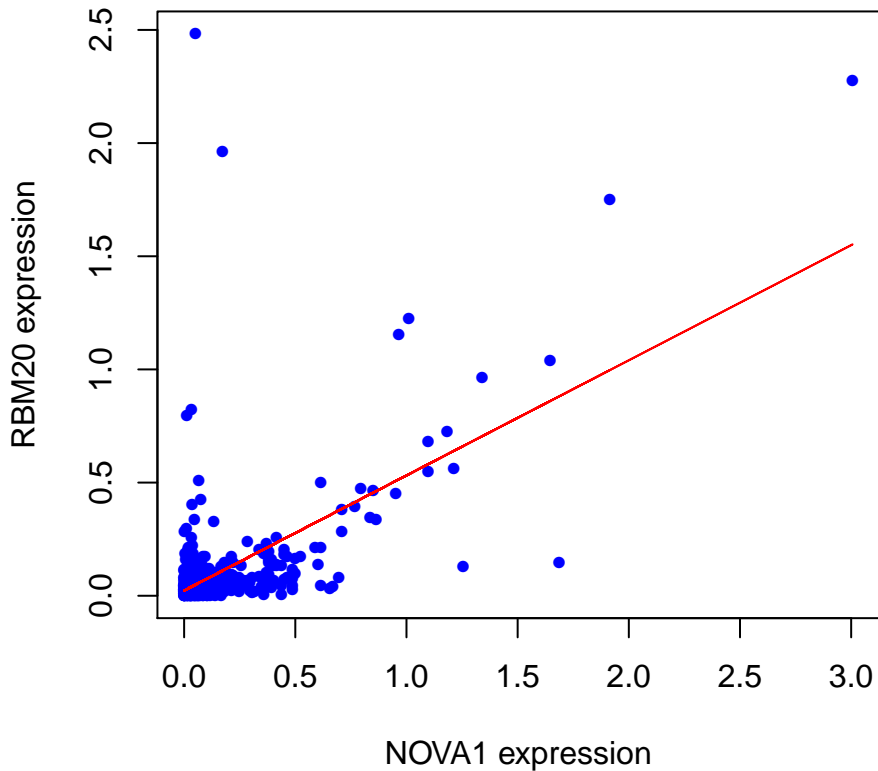

Supplement: Supplemental Information 6 [file peerj-09-11219-s006.zip › raw data/31.conexpression/17.cor/cor.NOVA1_RBM20.pdf]

**Cor=0.854 (p=2.149e-198)**

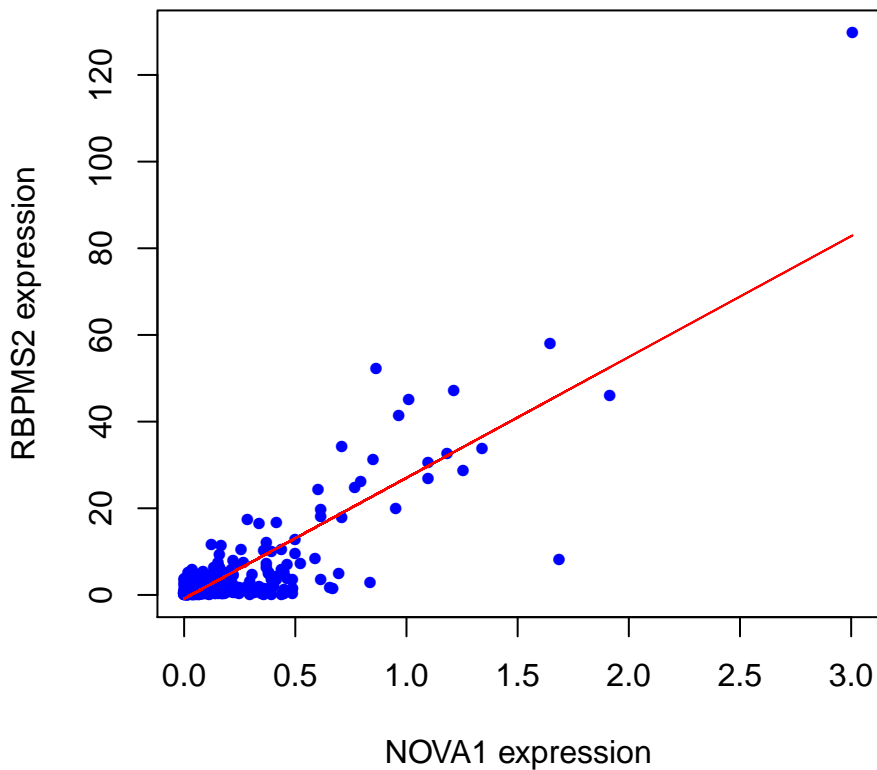

Supplement: Supplemental Information 6 [file peerj-09-11219-s006.zip › raw data/31.conexpression/17.cor/cor.NOVA1_RBPMS2.pdf]

**Cor=0.608 (p=4.938e-71)**

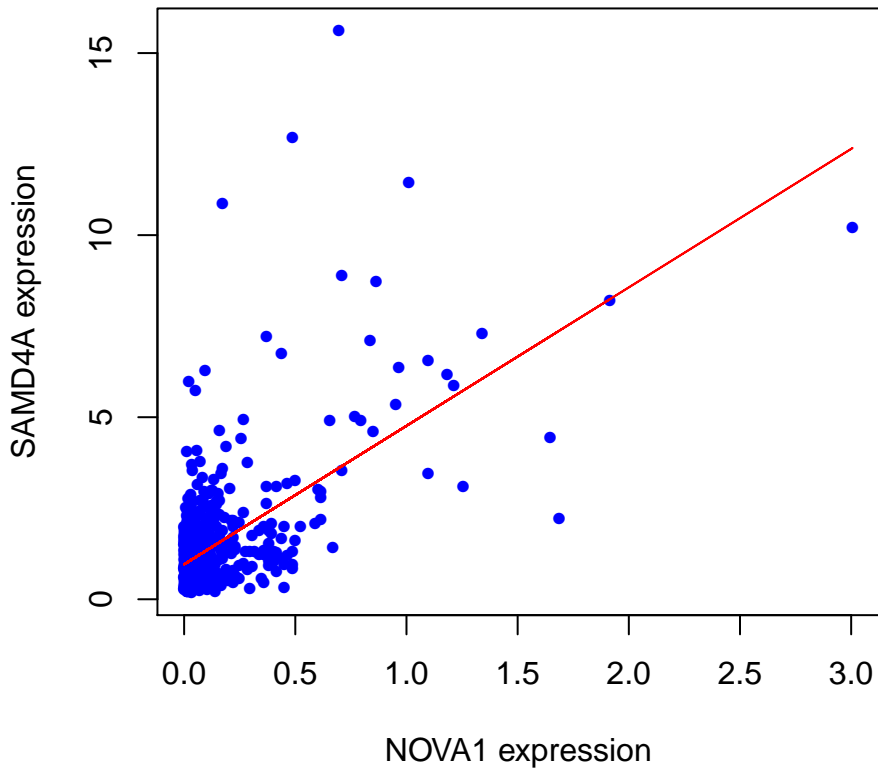

Supplement: Supplemental Information 6 [file peerj-09-11219-s006.zip › raw data/31.conexpression/17.cor/cor.NOVA1_SAMD4A.pdf]

**Cor=0.711 (p=7.902e-108)**

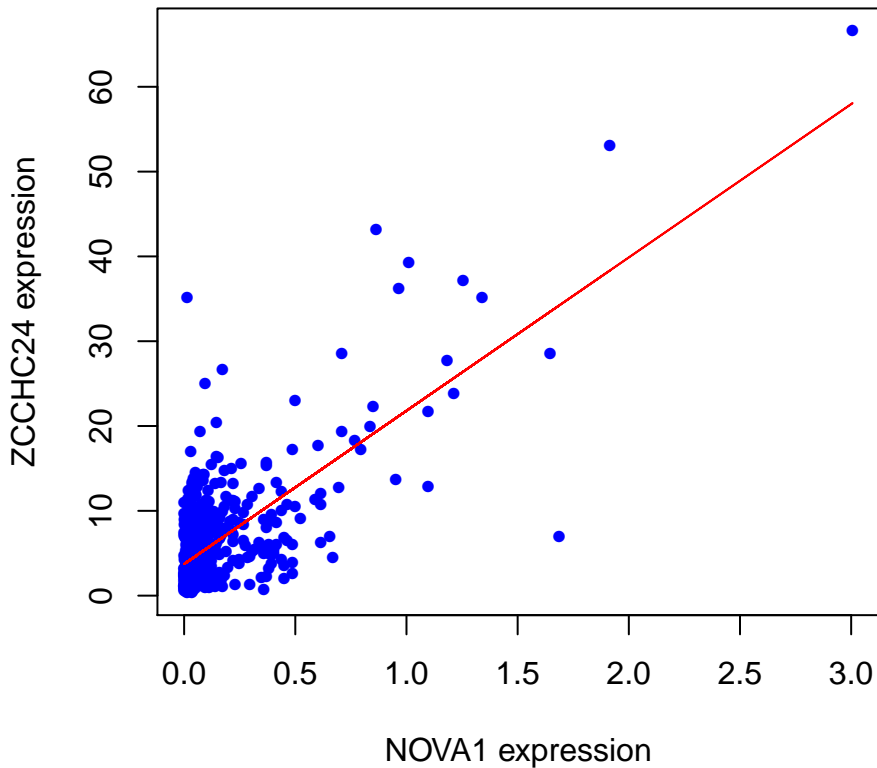

Supplement: Supplemental Information 6 [file peerj-09-11219-s006.zip › raw data/31.conexpression/17.cor/cor.NOVA1_ZCCHC24.pdf]

**Cor=0.637 (p=5.873e-80)**

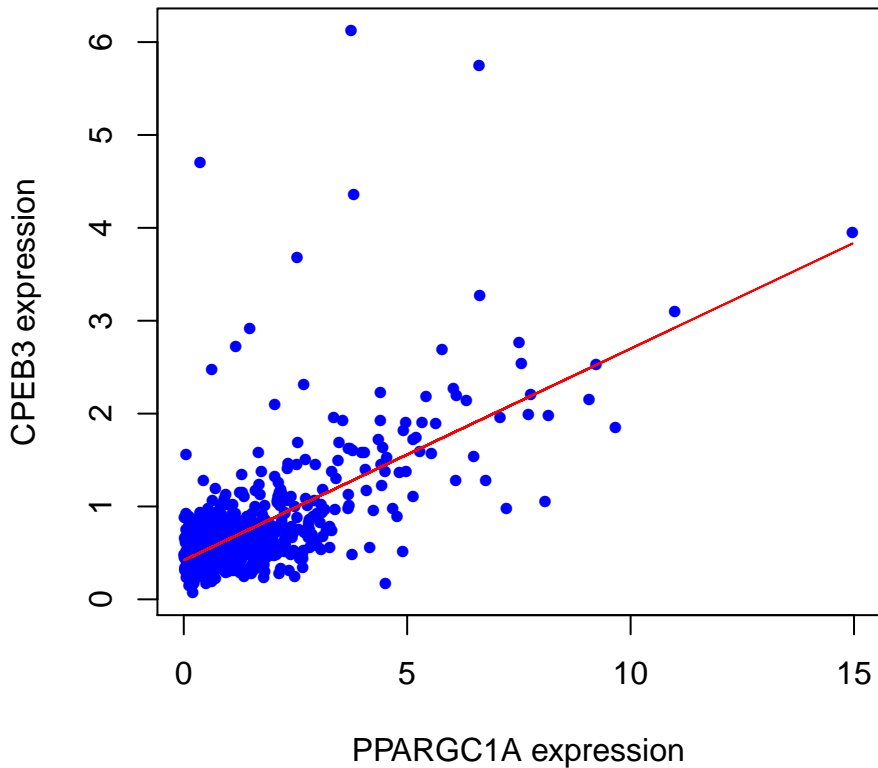

Supplement: Supplemental Information 6 [file peerj-09-11219-s006.zip › raw data/31.conexpression/17.cor/cor.PPARGC1A_CPEB3.pdf]

**Cor=0.659 (p=2.067e-87)**

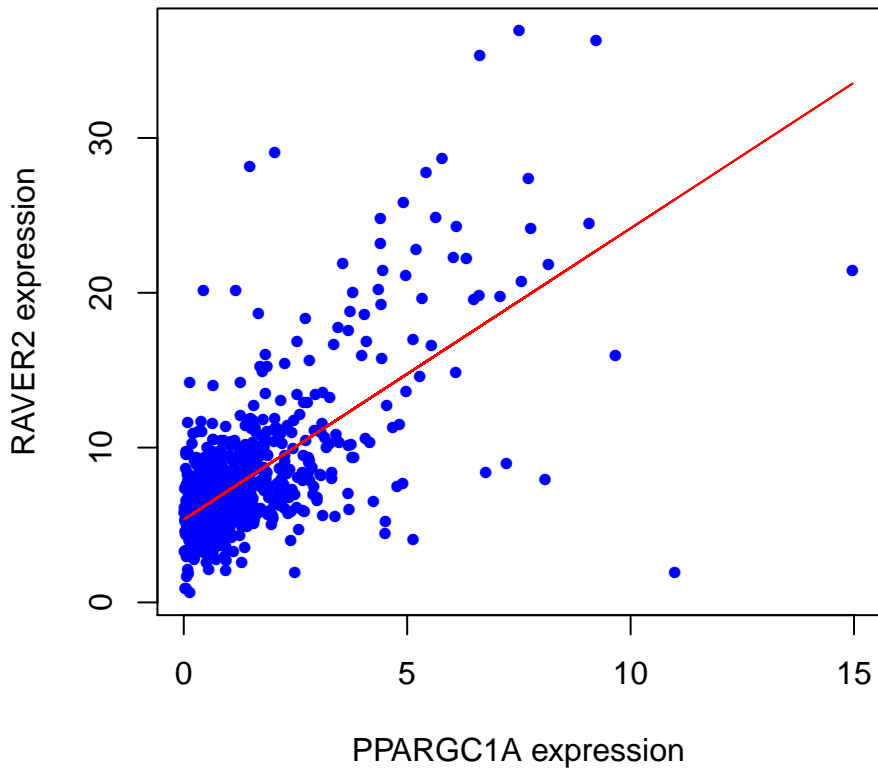

Supplement: Supplemental Information 6 [file peerj-09-11219-s006.zip › raw data/31.conexpression/17.cor/cor.PPARGC1A_RAVER2.pdf]

**Cor=0.702 (p=1.192e-103)**

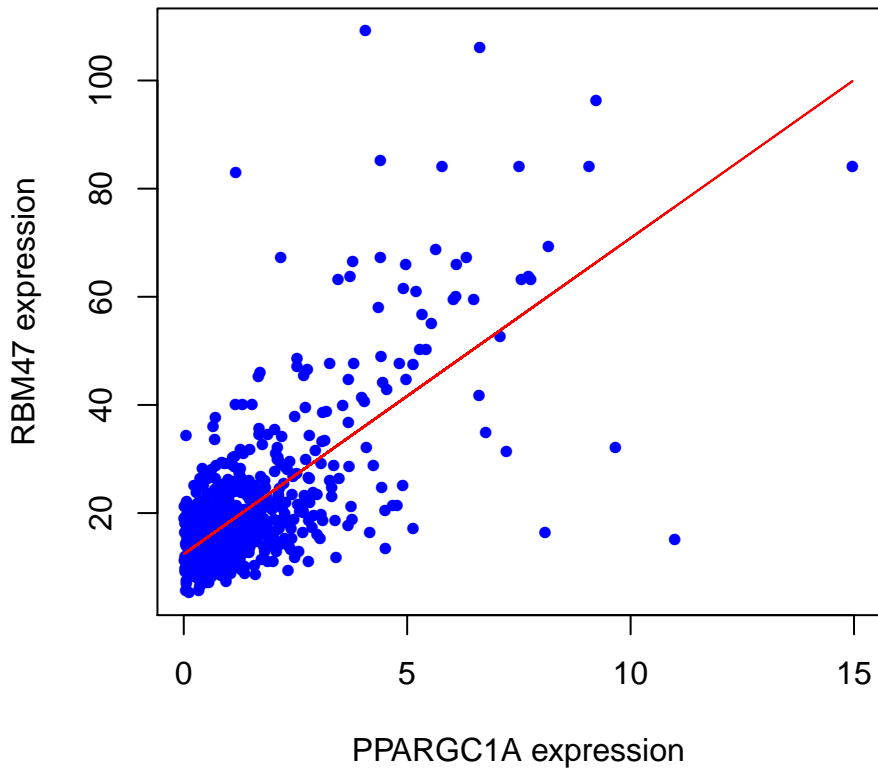

Supplement: Supplemental Information 6 [file peerj-09-11219-s006.zip › raw data/31.conexpression/17.cor/cor.PPARGC1A_RBM47.pdf]

**Cor=0.612 (p=2.186e-72)**

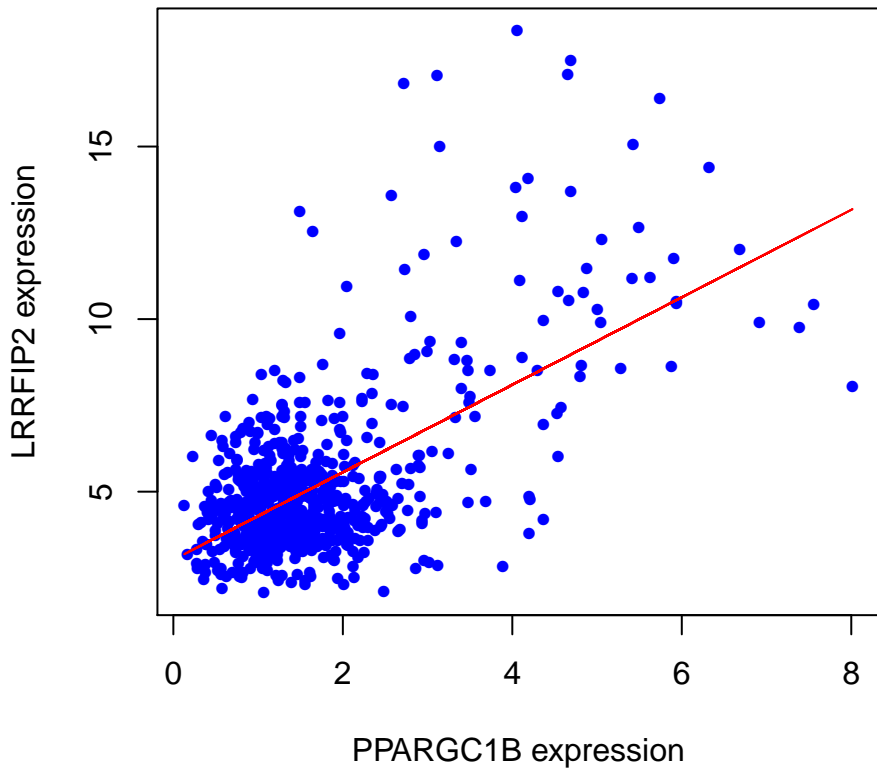

Supplement: Supplemental Information 6 [file peerj-09-11219-s006.zip › raw data/31.conexpression/17.cor/cor.PPARGC1B_LRRFIP2.pdf]

**Cor=0.627 (p=8.562e-77)**

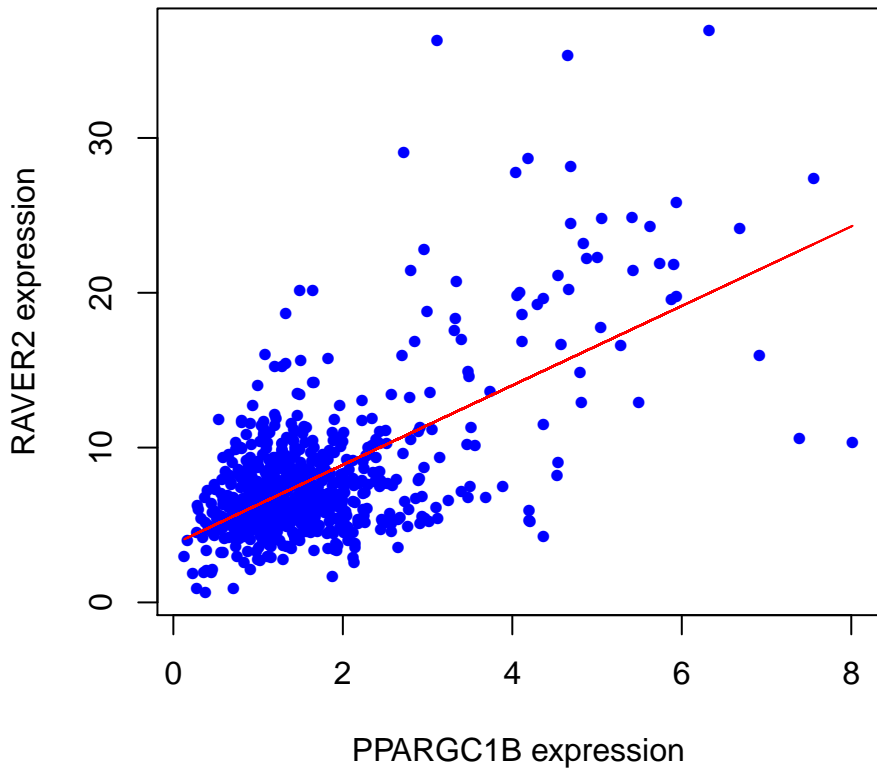

Supplement: Supplemental Information 6 [file peerj-09-11219-s006.zip › raw data/31.conexpression/17.cor/cor.PPARGC1B_RAVER2.pdf]

**Cor=0.711 (p=9.816e-108)**

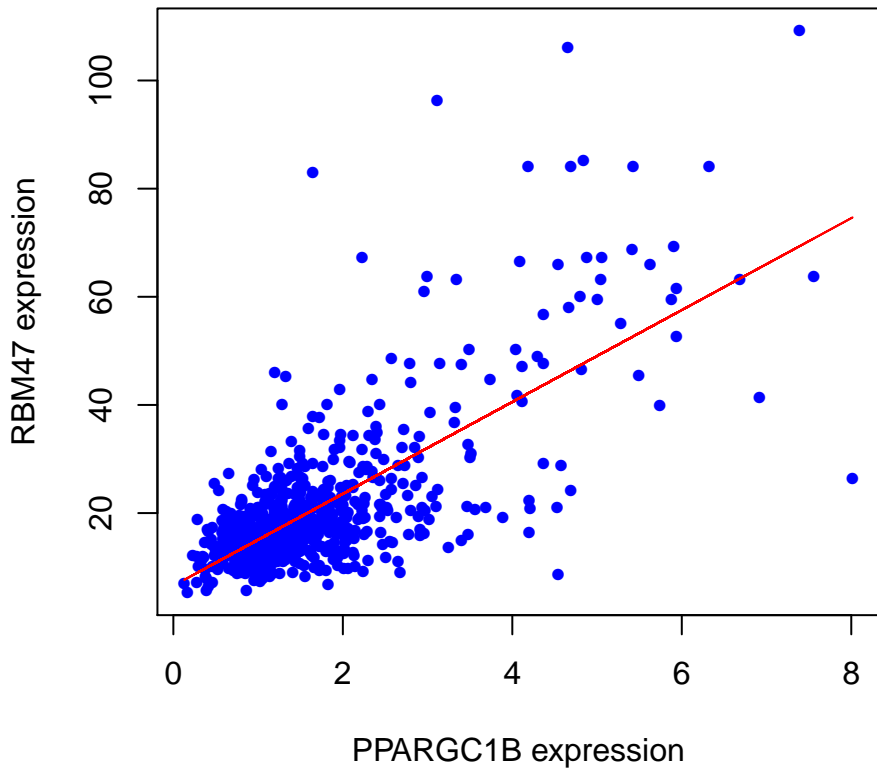

Supplement: Supplemental Information 6 [file peerj-09-11219-s006.zip › raw data/31.conexpression/17.cor/cor.PPARGC1B_RBM47.pdf]

**Cor=0.659 (p=2.498e-87)**

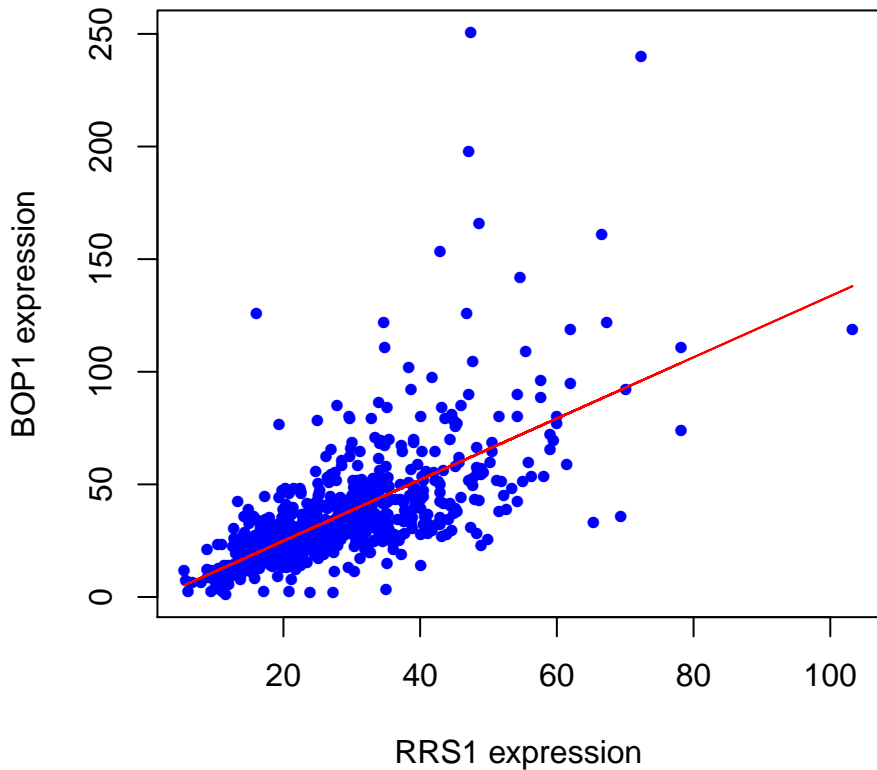

Supplement: Supplemental Information 6 [file peerj-09-11219-s006.zip › raw data/31.conexpression/17.cor/cor.RRS1_BOP1.pdf]
