# Supplementary material for: Construction and validation of an RNA-binding protein-associated prognostic model for colorectal cancer
Source: PeerJ. 2021 Apr 5;9:e11219. doi: 10.7717/peerj.11219 (PMC8029696; doi:10.7717/peerj.11219)
Supplement: Supplemental Information 6 [file peerj-09-11219-s006.zip › raw data/31.conexpression/18.ggalluvial/ggalluvial.pdf]

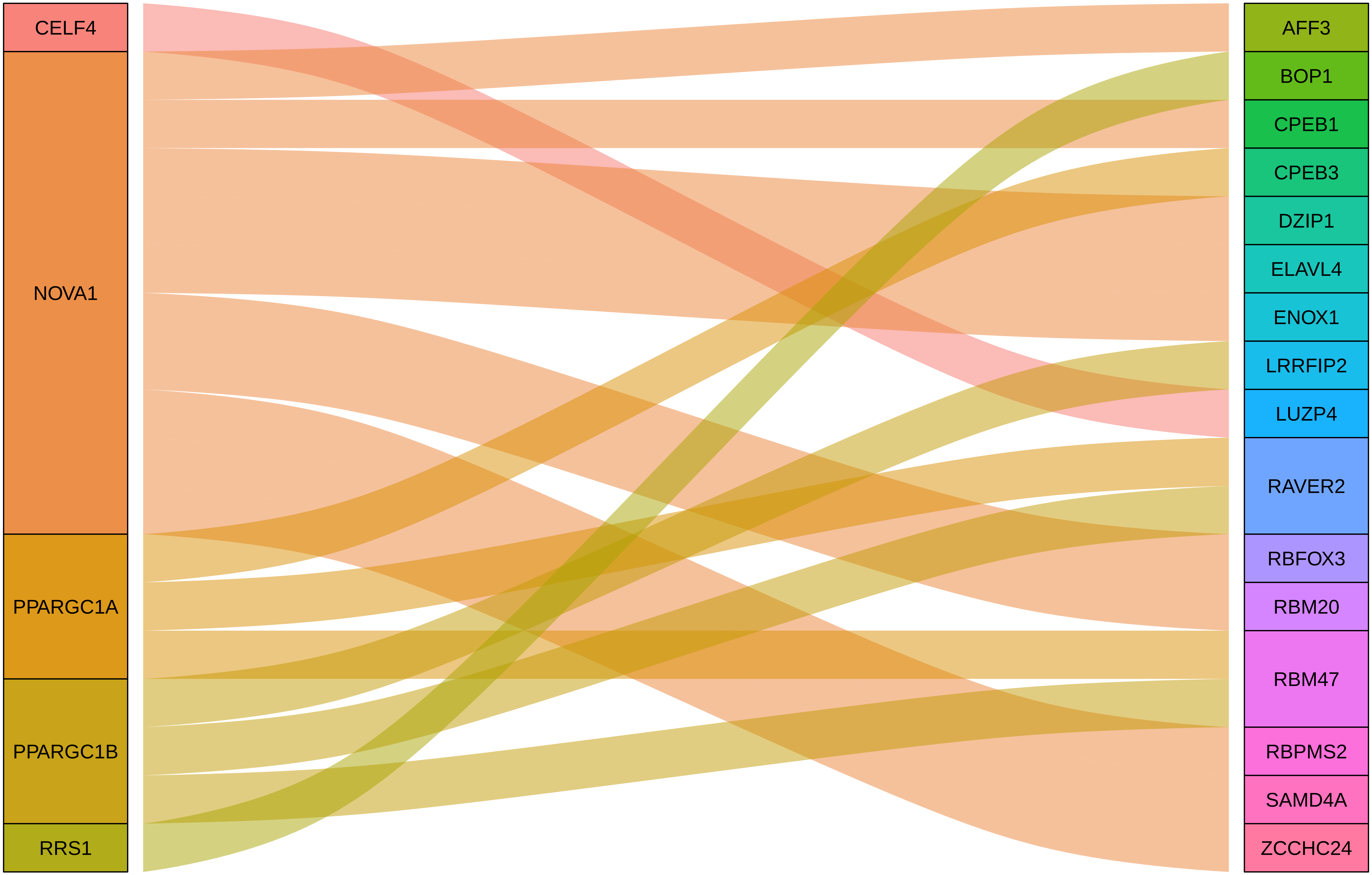
CELF4
NOVA1
PPARGC1A
PPARGC1B
RRS1
AFF3
BOP1
CPEB1
CPEB3
DZIP1
ELAVL4
ENOX1
LRRFIP2
LUZP4
RAVER2
RBFOX3
RBM20
RBM47
RBPMS2
SAMD4A
ZCCHC24
